# 16S microbiome analysis of microbial communities in distribution centers handling fresh produce

**DOI:** 10.3389/fmicb.2023.1041936

**Published:** 2023-07-12

**Authors:** Anna Townsend, Hendrik C. den Bakker, Amy Mann, Claire M. Murphy, Laura K. Strawn, Laurel L. Dunn

**Affiliations:** ^1^Department of Food Science and Technology, University of Georgia, Athens, GA, United States; ^2^Center for Food Safety, Department of Food Science and Technology, University of Georgia, Griffin, GA, United States; ^3^Department of Food Science and Technology, Virginia Tech, Blacksburg, VA, United States

**Keywords:** *Listeria*, built environment, next generation sequencing, food safety, supply chain

## Abstract

Little is known about the microbial communities found in distribution centers (DCs), especially in those storing and handling food. As many foodborne bacteria are known to establish residence in food facilities, it is reasonable to assume that DCs handling foods are also susceptible to pathogen colonization. To investigate the microbial communities within DCs, 16S amplicon sequencing was completed on 317 environmental surface sponge swabs collected in DCs (*n* = 18) across the United States. An additional 317 swabs were collected in parallel to determine if any viable *Listeria* species were also present at each sampling site. There were significant differences in median diversity measures (observed, Shannon, and Chao1) across individual DCs, and top genera across all reads were *Carnobacterium*_A, *Psychrobacter*, *Pseudomonas*_E, *Leaf454*, and *Staphylococcus* based on taxonomic classifications using the Genome Taxonomy Database. Of the 39 16S samples containing *Listeria* ASVs, four of these samples had corresponding *Listeria* positive microbiological samples. Data indicated a predominance of ASVs identified as cold-tolerant bacteria in environmental samples collected in DCs. Differential abundance analysis identified *Carnobacterium_A*, *Psychrobacter*, and *Pseudomonas_E* present at a significantly greater abundance in *Listeria* positive microbiological compared to those negative for *Listeria*. Additionally, microbiome composition varied significantly across groupings within variables (e.g., DC, season, general sampling location).

## 1. Introduction

Next generation sequencing (NGS) is providing guidance to some food safety efforts in the United States (US), especially outbreak traceback investigations (Ronholm et al., 2016; Jagadeesan et al., 2019; Stevens et al., 2022). In 2019, the US Centers for Disease Control and Prevention’s PulseNet laboratory network prioritized whole genome sequencing (WGS) over pulsed-field gel electrophoresis (PFGE) to identify the agents of foodborne illness outbreaks (Carleton, 2019; Ribot et al., 2019; Tolar et al., 2019). In addition to the ability to fingerprint bacterial pathogens, WGS also provides data to monitor phylogenetic relationships and address source attribution to prevent or mitigate future outbreaks (Ronholm et al., 2016; Mughini-Gras et al., 2018; Koutsoumanis et al., 2019). Microbiome sequencing, a type of NGS, is also being employed to analyze microbial communities in environmental and biological samples (Jagadeesan et al., 2019; Stevens et al., 2022). Microbiome sequencing can be completed with either 16S amplicon sequencing or shotgun metagenomic sequencing (Bharti and Grimm, 2021). Briefly, 16S amplicon sequencing is a targeted approach using hypervariable regions within the 16S rRNA gene contained in bacteria and archaea (Bharti and Grimm, 2021). Understanding bacterial communities in foods and the environments where foods are produced, processed, and handled can provide insights into the distribution of bacteria throughout the food system as well as antimicrobial and antibiotic resistance (Durso et al., 2012; Kovac et al., 2017; Lim et al., 2021).

Analysis of microbial communities for food safety applications has been completed in a handful of food-related environments and foods (Ottesen et al., 2013; Fang et al., 2015; Jarvis et al., 2015; Leonard et al., 2015, 2016; Dzieciol et al., 2016; Yang et al., 2016; Tan et al., 2019; Lim et al., 2021). For instance, shotgun metagenomic sequencing was used to detect pathogenic bacteria in environmental samples collected from groups of cattle as they moved across beef production chain stages (e.g., feedlots, cattle transport trucks, and holding pens; Yang et al., 2016); as well as to investigate the diversity and abundance of antibiotic resistance genes in greenhouse soils (Fang et al., 2015). Additionally, 16S amplicon sequencing of the V4 domain was used to determine the composition of microbiota in fruit processing environments (Tan et al., 2019). Moreover, bacterial communities within drain water and drain biofilms in cheese processing facilities were examined using pyrosequencing of 16S rRNA genes (Dzieciol et al., 2016), while 16S amplicon sequencing was used to identify a cross-contamination pathway of bacteria in a foodservice facility (Lim et al., 2021). Furthermore, bacterial foodborne pathogen (e.g., *Salmonella*, Shiga toxin-producing *E*. *coli*) detection in cilantro, spinach, and tomatoes has been completed using a metagenomic approach (Ottesen et al., 2013; Jarvis et al., 2015; Leonard et al., 2015, 2016).

Metagenomic examination of food facilities, such as processing facilities or DCs, is still lacking based on currently published data (Stevens et al., 2022). One study has examined the relationship between the microbiome and *Listeria* presence in a meat processing facility (Belk et al., 2022). Certain microorganisms were linked to *Listeria* presence in specific rooms of the facility, and *Listeria* spp. were also distributed by room function (Belk et al., 2022). There is also limited research on microbial presence within DCs that handle food (Townsend et al., 2021, 2022). Only one study (Townsend et al., 2022) has explored factors affecting *Listeria* presence in DCs handling fresh produce, and found that 5% of environmental samples collected contained isolatable *Listeria* species.

In the current study, 16S amplicon sequencing of environmental samples was completed to investigate microbial communities in 18 DCs across the United States. The objectives include examining microbiome diversity within and between DCs, determining the existence of any relationships between *Listeria* spp.-positive samples and abundant taxa, and identifying top taxa across all DCs.

## 2. Materials and methods

### 2.1. Sample collection

Between December 2019 and March 2021, 18 DCs across the contiguous United States were visited once per DC to collect environmental surface samples (Townsend et al., 2022). Samples were collected using sterile sponge sticks pre-moistened with 10 mL of Dey-Engley broth (3 M, St. Paul, MN, United States). The hygienic zone concept was used to classify sample sites based on likelihood of introducing microbiological hazards to product in the DCs. Only non-food contact surfaces were swabbed (40 cm × 40 cm areas); therefore, no zone 1 surfaces were examined. Zone 2 surfaces were also not examined as results from Townsend et al. (2022) indicated increased *Listeria* prevalence in DCs on zone 3 and 4 surfaces. These locations are also known harborage sites of *Listeria* spp. in food-related environments (Carpentier and Cerf, 2011; Hoelzer et al., 2011). Two sponge swabs were collected in adjacent sampling areas: one for microbiological analysis and the other for 16S amplicon analysis (*n* = 317 for each analysis). Additional metadata, including sampling site location and other DC characteristics (e.g., geographic region, and state), were recorded (Supplementary Table S1 in Supplementary material).

### 2.2. Microbiological and 16S amplicon sample processing

All sponge swabs were returned to their original bags with the sticks removed and kept on ice during shipment. Only sponges used for microbiological analysis were processed using the U.S. Food and Drug Administration’s Bacteriological Analytical Manual (FDA-BAM) for *Listeria* detection in environmental samples (Hitchins et al., 2017). Upon arrival to the laboratory, 16S amplicon sponges were stored at −20°C. Prior to processing, sponges were thawed and kept on ice. 16S amplicon sponges were processed according to the methodology in Tan et al. (2019) and did not undergo an enrichment. Briefly, 90 mL of 1X phosphate-buffered saline (PBS) solution (Thermo Fisher Scientific, Waltham, MA, United States) was added to each sponge sample bag. Bags were homogenized in a Stomacher Model 400 Circulator Lab Blender (Seward, Worthing, West Sussex, United Kingdom) at 230 RPM for 7 min and approximately 45 mL of homogenate was poured into a 50 mL sterile conical tube. Tubes were stored at −80°C until DNA extraction. The DNeasy PowerSoil Pro Kit (Qiagen, Venlo, Netherlands) was used for DNA extraction following the manufacturer’s instructions; extractions were stored at −80°C until 16S amplicon sequencing. Sample processing was completed at two laboratories, one at Virginia Tech and the other at the University of Georgia.

### 2.3. 16S amplicon sequencing

Extracted DNA was transported on dry ice to the Center for Food Safety in Griffin, GA and kept at −20°C until use. For each extracted DNA sample, double-stranded DNA (dsDNA) concentration was determined using a Qubit fluorometer (Invitrogen, Waltham, MA, United States) with the Qubit dsDNA High Sensitivity Assay Kit. Sequencing preparation was completed using the 16S Metagenomic Sequencing Library Preparation method from Illumina (San Diego, CA, United States). Sample libraries were prepared using amplicon PCR to target the V3 and V4 regions of the 16S rRNA gene (Illumina, 2021) with forward primer 5′-TCGTCGGCAGCGT CAGATGTG TATAAGAGACAGCCTACGGGNGGCWGCAG-3′ and reverse primer 5′-GTCTCGTGGGCTCGGAGA TGTGTATAAGAGACAG GACTACHVGGGTATCTAATCC-3′. Qubit dsDNA concentrations were determined once more after amplicon PCR. AMPure XP beads were used to purify amplicon libraries prior to sequencing on the Illumina MiSeq platform (Illumina, San Diego, CA, United States) to produce 2 × 300 bp paired end reads. For each amplicon PCR a negative control was included, for a total of three negative controls per sequence run.

### 2.4. Bioinformatic analysis

**Figure 1:**
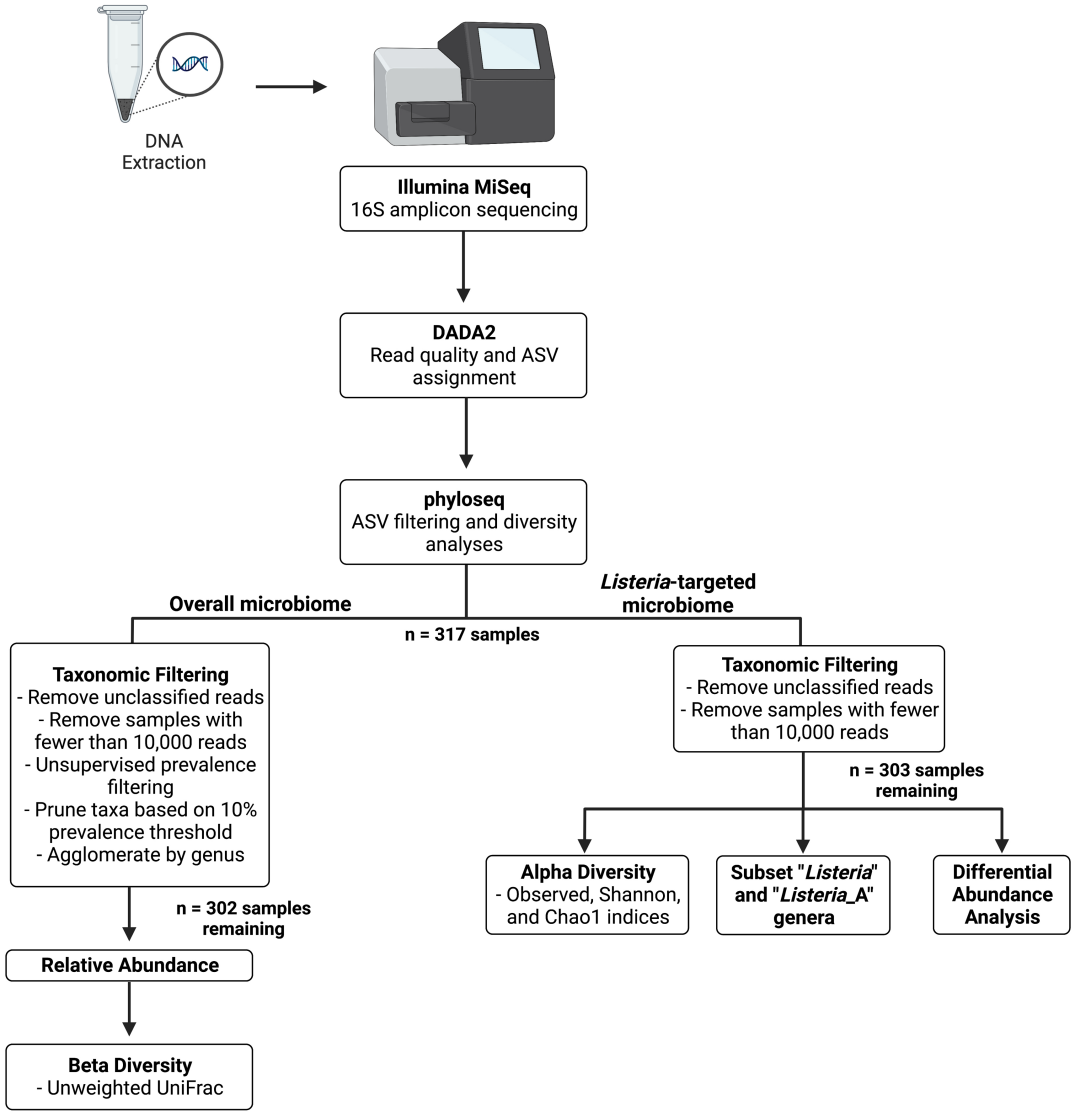
Flow diagram of 16S amplicon sequencing workflow.

Figure 1 provides a flowchart of bioinformatic analyses. Raw sequence reads in FASTQ format were processed and analyzed using *dada2* (v1.22.0; Callahan et al., 2016a). Reads were filtered and trimmed using the filterAndTrim function within *dada2*, with a truncLen of 260, 220, and a maxEE of 2.5 to remove low-quality reads. Pooling was also completed on forward and reverse reads with *dada2* using pseudo-pooling to assist in rare amplicon sequencing variant (ASV) detection. Taxonomy was assigned using the Genome Taxonomy Database (GTDB; release 95), and frequency tables and taxonomic classifications were exported to R’s serialized format for further analysis using *phyloseq* (v1.38.0; McMurdie and Holmes, 2013) in RStudio (v4.0.0). Read characteristics after processing with *dada2* were investigated using the summarize_phyloseq function in the *microbiome* package (v1.16.0; Lahti and Shetty, 2017). Metadata, including sample and DC characteristics, were also included in the phyloseq object.

Two workflows were used to analyze the (1) *Listeria*-targeted microbiome and (2) the overall microbiome (refer to Figure 1). This was completed because of the relatively low abundance of *Listeria-*identified reads, which were not maintained after taxonomic filtering with a 10% prevalence threshold. A 10% prevalence threshold was also used to capture high abundance taxa within each phylum and reduce those that did not appear to have a meaningful biological contribution. This cut-off was determined by plotting prevalence by total abundance of taxa within each phylum, as described in Callahan et al. (2016b). For maintaining *Listeria-*specific reads, only unobserved taxa (i.e., taxa without matched ASVs) and samples with fewer than 10,000 reads were removed. The threshold of 10,000 reads was chosen after analysis of the negative PCR controls, which showed a maximum number of reads below 10,000. Singletons, which are individual ASVs matched with only one read, were maintained in the *Listeria-*targeted microbiome to evaluate richness in alpha diversity indices. After these processing steps, 303 samples remained (317 original samples) for determining the relative abundance and subsetting reads with *Listeria*-identified ASVs for further analysis. Alpha diversity measures (observed, Shannon, and Chao1) were calculated after removing unobserved taxa and samples containing less than 10,000 reads. Kruskal-Wallis one-way ANOVA with Dunn’s *post hoc* test was used to compare alpha diversities across metadata variables (e.g., general location, season). NCBI BLAST (Altschul et al., 1990) was used to investigate ASVs of interest in the data.

For the overall microbiome, reads with unobserved taxa were removed. Taxonomic filtering was completed using methods from Callahan et al. (2016b). After pruning taxa, samples with fewer than 10,000 reads were also removed. Taxa were agglomerated by genus prior to determining relative abundance, leaving 302 samples for beta diversity analyses. The *msa* (v1.26.0; Bodenhofer et al., 2015) package was used to complete a multiple sequencing alignment with method “muscle” using sequences extracted from the *phyloseq* object, and *phangorn* (v2.9.0; Schliep, 2011) was used to build a maximum likelihood phylogenetic tree. Beta diversity was completed using the *phyloseq* distance function with the unweighted UniFrac method (Lozupone and Knight, 2005). Analyses for determining variance and composition among unweighted UniFrac distances grouped by variable were completed with functions betadisper and adonis2 (PERMANOVA) from the *vegan* package (v2.6-2; Oksanen et al., 2020). Plots were produced using *ggplot2* (v3.3.6; Wickham, 2016), or with functions from previously mentioned packages. *phylosmith* (v1.0.6; Smith, 2022) was used for determining common taxa among samples grouped by variable levels. *DESeq2* (v1.34.0; Love et al., 2014) was used for differential abundance analysis including a Wald test with a parametric fit type and significance level of 0.01. Additionally, ANCOM-BC (v2.0.2; Lin and Peddada, 2020) was used to infer differentially abundant ASVs with a significance level of 0.01.

## 3. Results and discussion

### 3.1. Sample characteristics

For microbiological samples, 18 out of 303 (5.9%) contained isolatable *Listeria* species through selective culture enrichment and plating. Sample characteristics, including corresponding DC, general and primary locations, state, geography, season, zone, site dryness, cleaning type, and *Listeria* spp. presence can be found in Supplementary Table S1 (Supplementary material). The average number of one sample type collected per DC was approximately 17, with DCs D and R containing the fewest and greatest number of samples at 5 and 34, respectively. Table 1 provides the number of samples per primary location within each general location.

**Table 1:** Number of samples and percentage by general location collected (n = 303) per general and primary locations.

General location	Primary location^a^				Total
	Barrier	Cleaning	Floor	Wall	
50F room	6 (11.5)	11 (21.2)	35 (67.3)	-	52
Banana rooms	1 (14.3)	-	6 (85.7)	-	7
Cleaning area	-	16 (76.2)	5 (23.8)	-	21
Cold storage	13 (13.4)^b^	26 (26.8)	58 (59.8)	-	97
Equipment storage	-	5 (83.3)	1 (16.7)	-	6
Merge	-	-	8 (100)	-	8
Receiving	8 (18.6)	10 (23.3)	24 (55.8)	1 (2.3)	43
Shipping	2 (14.3)	5 (35.7)	5 (35.7)	2 (14.3)	14
Receiving/shipping	8 (14.5)	11 (20)	33 (60)	3 (5.5)	55
Total	38	84	175	6	303

a Dashes indicate no samples were collected at that location.

b One sample taken in cold storage on a barrier was not included in the overall microbiome workflow after taxonomic filtering.

Six samples were collected on zone four surfaces (e.g., floors distant from produce storage/transit areas), while the remaining 297 samples were collected on zone three surfaces (e.g., cleaning-related surfaces, floors in closer proximity to produce storage/transit areas). Sample site dryness was also recorded, with 271 (89.4%) samples collected on dry surfaces and 32 (10.6%) on wet surfaces. For cleaning type, most samples were from sites that experienced dry and wet cleaning (148/303, 48.8%), followed by wet cleaning only (68/303, 22.4%), no cleaning (61/303, 20.1%), and dry cleaning only (26/303, 8.58%).

Samples were collected primarily in Michigan (69/303, 22.8%), followed by Pennsylvania (43/303, 14.2%), Maryland (33/303, 10.9%), New York (31/303, 10.2%), Wisconsin (28/303, 9.24%), Massachusetts (27/303, 8.91%), Florida (24/303, 7.92%), Texas (13/303, 4.29%), Georgia (12/303, 3.96%), North Carolina (9/303, 2.97%), Ohio (9/303, 2.97%), and Kentucky (5/303, 1.65%). Geographically, 134 (44.2%) samples were collected in the Northeast, 111 (36.6%) in the Midwest, and 58 (19.1%) in the South, as defined by U.S. Department of Agriculture Agricultural Research Service (USDA ARS) regions. Most samples were collected in winter (184/303, 60.7%), followed by spring (65/303, 21.5%), and fall (54/303, 17.8%).

16S amplicon read characteristics, including statistical information such as average reads per sample and number of singletons, are presented in Supplementary Table S2 (Supplementary material).

### 3.2. *Listeria-*targeted microbiome

#### 3.2.1. Alpha diversity by individual DC

**Figure 2:**
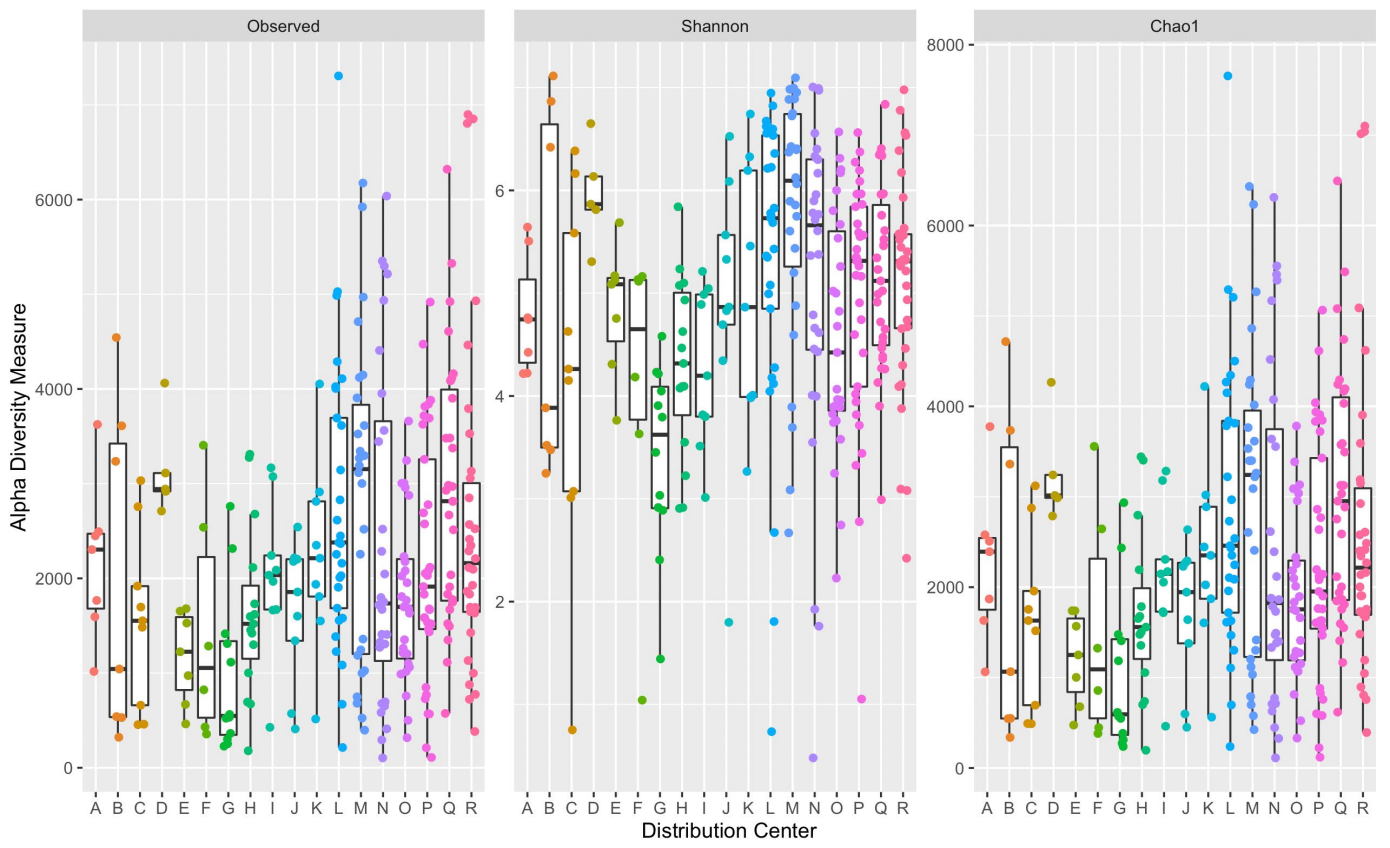
Side-by-side boxplots of observed, Shannon, and Chao1 alpha diversity indices across individual DCs. Boxplots provide median, first and third quartiles, and max and minimum (whiskers) of alpha diversity measures. Each point represents a single sample’s corresponding alpha diversity measure.

A comparison of alpha diversity indices per sample grouped by corresponding DC are presented in Figure 2. There was a significant difference in median diversity measures between individual DCs across all alpha diversity indices (observed: *p* = 6.62 × 10^−5^, Shannon: *p* = 3.44 × 10^−6^, and Chao1: *p* = 6.75 × 10^−5^). Dunn’s *post hoc* analysis across all alpha diversity indices indicated significant differences (*p* ≤ α/2) in median values between DCs G and L, G and M, and G and Q. Significant differences in median Shannon index values were also observed between G and D, H and M, N and G, R and G, and O and M. DC M had the greatest median Chao1 index (3,242) suggesting a richer microbial community compared to that in the other DCs. DC G had the lowest median Chao1 index (593). Some variation across alpha diversity measures may be due to the number of samples collected within each DC. The average number of samples collected per DC was approximately 17, with DCs D and R containing the fewest and greatest number of samples at 5 and 34, respectively.

#### 3.2.2. Alpha diversity by United States geographical location

**Figure 3:**
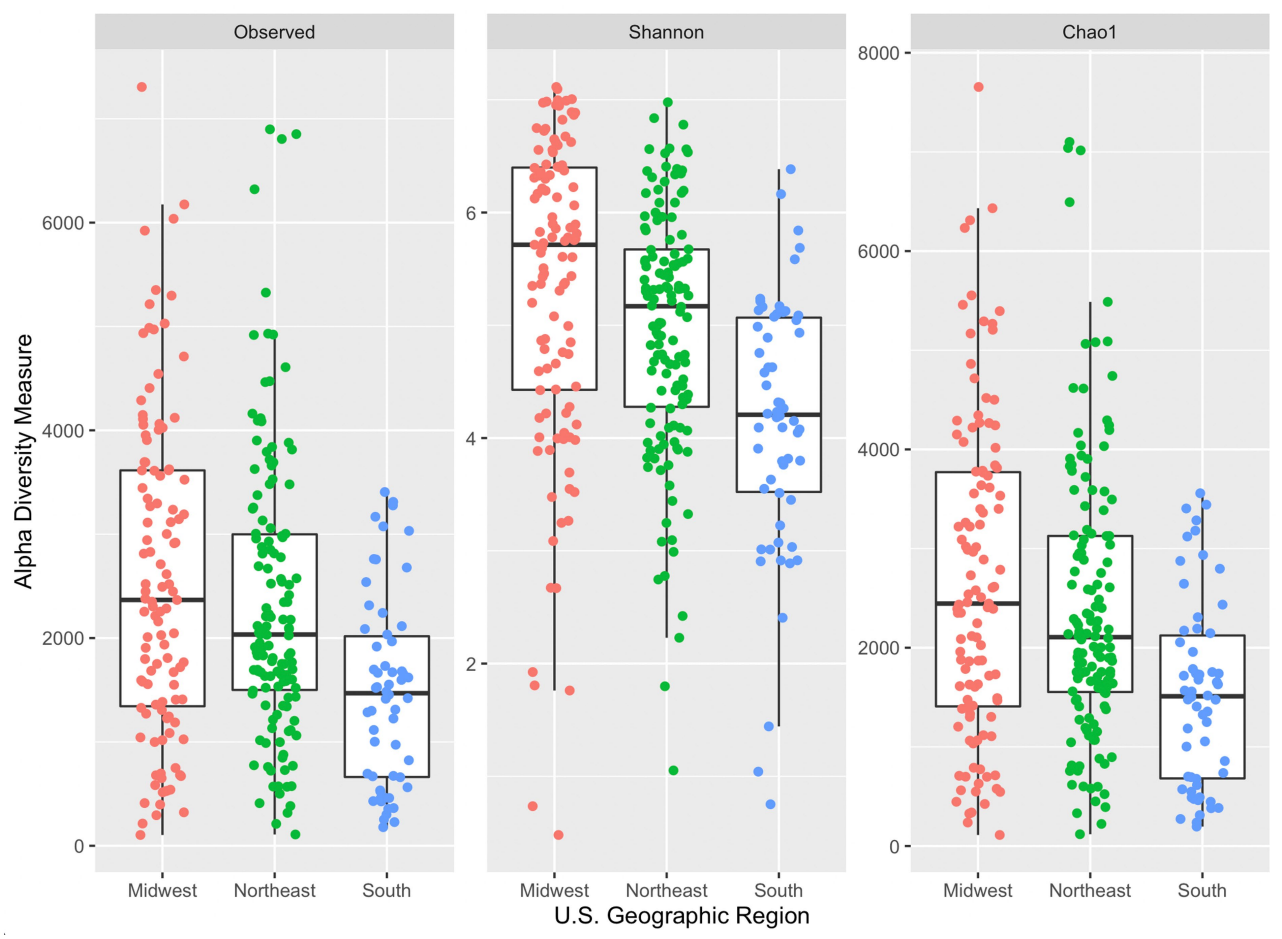
Side-by-side boxplots of observed, Shannon, and Chao1 alpha diversity indices across United States geographic regions.

All three alpha diversity indices also indicated a significant difference in median diversity measure between samples grouped by U.S. geographic region (observed: *p* = 5.33 × 10^−6^, Shannon: *p* = 4.97 × 10^−9^, and Chao1: *p* = 5.57 × 10^−6^; Figure 3). Dunn’s *post hoc* comparison indicated significant differences between Shannon’s diversity indices between all geographic region pairs (South and Midwest, South and Northeast, and Northeast and Midwest). However, significant differences were only seen in observed and Chao1 diversity indices between South and Midwest and South and Northeast. The Midwest region had the greatest median Chao1index (2,445), followed by the Northeast (2,106), and South (1,510). Generally, median diversity measures decrease from Midwest to Northeast to South across all indices. It is not evident why this trend appears across regions. While there was a significant difference observed across median diversity measures, the distribution of samples across each region is similar (especially within the Shannon index). Sample size may also contribute to differences among alpha diversity indices across region, as only 58 samples were collected in the South, followed by 111 in the Midwest, and 134 in the Northeast.

#### 3.2.3. Alpha diversity by dryness

**Figure 4:**
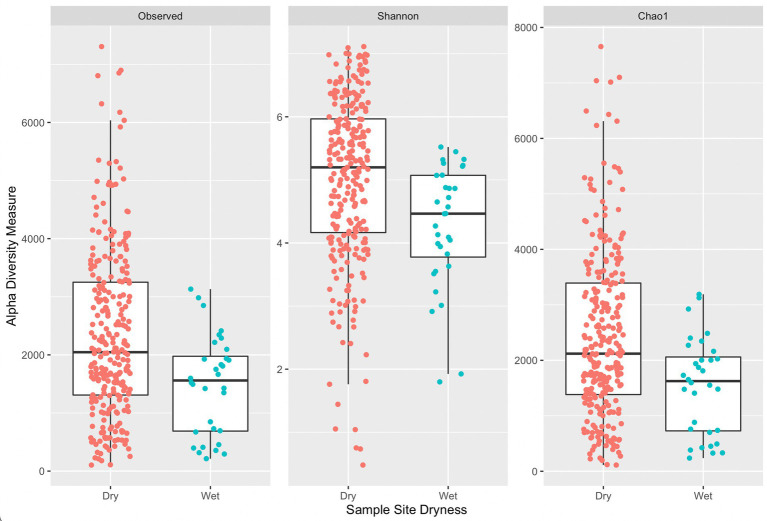
Side-by-side boxplots of observed, Shannon, and Chao1 alpha diversity indices across sample site dryness.

Figure 4 provides a comparison of alpha diversity indices per sample grouped by sample site dryness (wet or dry). Wilcoxon rank sum tests with continuity correction across all three indices indicated a significant difference between median alpha diversity measures between dry and wet sample sites (observed: *p* = 0.0015, Shannon: *p* = 0.00036, Chao1: *p* = 0.0014). Generally, samples collected on wet surfaces had a lower median diversity measure (across all three indices) compared to samples collected on dry surfaces. It is unexpected that samples collected on dry surfaces would have a greater diversity of ASVs, indicating a greater diversity of microorganisms, as a dry surface would not be favorable for microbial residence. An exception would be if wet surfaces contained antimicrobial agents, such as sanitizers, or other compounds that could lead to DNA degradation, that could lead to fewer ASVs.

#### 3.2.4. Alpha diversity by *Listeria* presence

**Figure 5:**
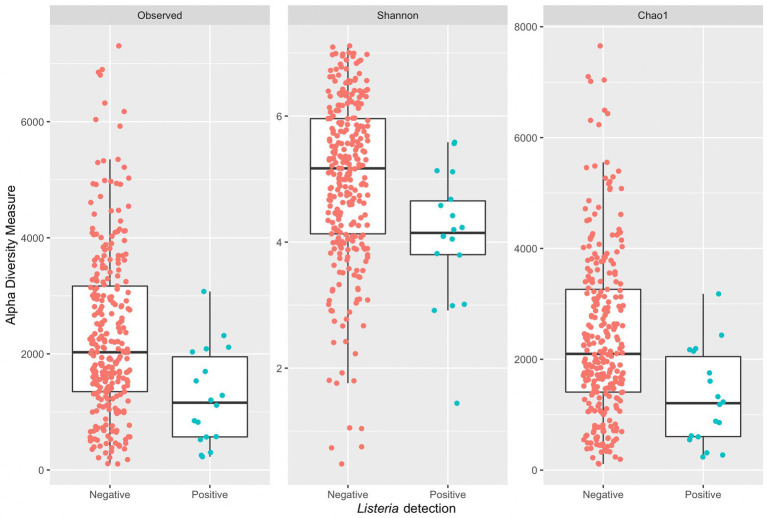
Side-by-side boxplots of observed, Shannon, and Chao1 alpha diversity indices across *Listeria* positive and negative microbiological samples.

A Wilcoxon rank sum test with continuity correction indicated a significant difference in median diversity values between samples grouped by positive or negative *Listeria* microbiological samples across all alpha diversity indices (observed: *p* = 0.0011, Shannon: *p* = 0.0018, and Chao1: *p* = 0.0011; Figure 5). Samples with corresponding microbiological samples negative for *Listeria* presence had a greater median Chao1 value (2,097) compared to those positive for *Listeria* presence (1,207). Across all indices, samples positive for *Listeria* have a lower median measure compared to those negative for *Listeria*. It is suspected that small sample size might contribute to a lower median measure in 16S amplicon samples with corresponding microbiological samples positive for *Listeria*. Furthermore, box plots (Figure 5) corresponding to *Listeria* positive samples completely overlap with those for *Listeria* negative samples, so it is not unreasonable to assume an increase in sample size might change the distribution of alpha diversity measures.

#### 3.2.5. *Listeria* abundance

**Figure 6:**
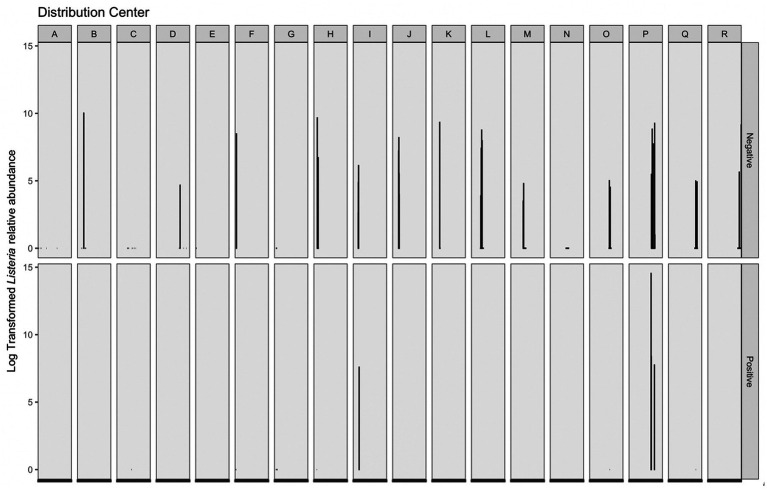
Log transformed *Listeria* relative abundance (left *y*-axis) grouped by corresponding distribution center (top *x*-axis) and *Listeria* microbiological sample (right *y*-axis). There were 285 *Listeria* negative microbiological samples, 18 *Listeria* positive samples; however, only 39 total samples contained log transformed relative abundance values greater than 0–4 of these samples were *Listeria* positive and 35 were *Listeria* negative.

*Listeria*-identified ASVs were only present in the phyloseq object when the prevalence threshold was equal to approximately 4%; therefore, a second workflow was used to maintain *Listeria*-identified ASVs without compromising on analysis of the overall microbiome. Across 16S amplicon samples, a greater log transformed *Listeria* relative abundance was observed in corresponding microbiological samples negative for *Listeria* (Figure 6). The range of log transformed relative abundance across samples was 0–14.54. Thirty-nine samples contained log transformed relative abundance values greater than zero; four of these samples had corresponding *Listeria* positive microbiological samples, while the remaining 35 corresponded to *Listeria* negative microbiological samples. Across those samples, the average log transformed relative abundance was 6.42. The sample with the greatest log transformed relative abundance (14.54) was from DC P and corresponded with a *Listeria* positive microbiological sample. This sample was taken on a wet floor within a cleaning area. The other three samples with *Listeria* positive microbiological samples and log transformed relative abundance greater than zero were collected on cleaning equipment within a cleaning area (DC P), on the floor in cold storage (DC P), and on the floor in a “10°C” (50°F) room (DC I). Fourteen 16S amplicon samples with log transformed *Listeria* relative abundance of zero had corresponding *Listeria* positive microbiological samples. Additionally, the majority of 16S amplicon samples with log transformed relative abundance values greater than zero had corresponding microbiological samples negative for *Listeria*. Therefore, microbiome samples taken adjacently to *Listeria* positive microbiological samples are not necessarily indicative of *Listeria* presence within a given sampling site. This have been a limitation of this study, as single sponges were not analyzed for both isolatable *Listeria* and the overall microbiome. While microbiome approaches can be used as a tool to understand microbial communities within environments, they cannot replace culture-based methods as indicators of live bacteria presence.

Five *Listeria*-identified ASVs were present in sample reads. Based on NCBI BLAST queries of ASV sequences, three ASVs were putatively classified within *Listeria sensu stricto* group, one was putatively within the *Listeria sensu lato* group, and the last was undetermined (Supplementary Table S3 in Supplementary material). Across samples that contained isolatable *Listeria*, their corresponding microbiome samples contained two common *Listeria*-identified ASVs (one putative *sensu stricto* and one putative *senso lato*). However, for those samples that were negative for isolatable *Listeria*, their corresponding microbiome sample contained five common *Listeria*-identified ASVs. Therefore, there were more *Listeria*-identified ASVs in microbiome samples that did not have corresponding *Listeria* positive microbiological samples. It is possible that this may explain the greater overall *Listeria* read abundance seen in *Listeria* negative samples (Figure 6). However, there was also a greater number of samples with corresponding microbiological samples negative for *Listeria* (*n* = 285) compared to that of positive samples (*n* = 18), which may also influence the total number and consequent abundance of *Listeria* reads.

#### 3.2.6. Differential abundance analysis

*DESeq2* and ANCOM-BC were used to determine if there were any significantly different relative abundances of taxa among corresponding microbiological samples that were negative (reference) or positive (contrast) for *Listeria* (Supplementary Table S4 in Supplementary material). The results of the *DESeq2* analysis showed only two genera, *Psychrobacter* and *Pseudomonas_E*, were present at a significantly greater abundance in microbiological samples positive for *Listeria* compared to those negative for *Listeria*. Log2 fold change values for *Psychrobacter* and *Pseudomonas_E* were 4.16 and 2.84, respectively. The remaining taxa had negative log_2_ fold change values, indicating they had a significantly lower abundance in microbiological samples positive for *Listeria* compared to those that were negative. The top three genera with a significantly lower abundance in microbiological samples positive for *Listeria* were *Pseudomonas_E*, *Lacibacter*, and *Bradyrhizobium* with log_2_ fold change values of −26.46, −26.05, and −25.71, respectively. The results of the ANCOM-BC analysis showed a large number of ASVs (23,056) having significantly higher or lower abundance in *Listeria* positive samples. Log_2_ fold change values higher than 1 or lower than −1 were only found for 179 ASVs. Only two ASVs mapping to *Carnobacterium* and *Psychrobacter*, had positive Log_2_ fold change values of 2.00 and 1.87, respectively. Of the 177 ASVs with a log_2_ fold change of −1 or lower, there were five ASVs with a log_2_ fold change lower than −2, matching partial 16S sequences of *Microbacterium*, *Elkelangia*, *Jeotgalibaca*, *Polaromonas*, and an ASV with a high BLAST similarity (100% identity, E-value 0.0) to *Cryobacterium* species (i.e., *C*. *soli*, *C*. *zongtaii*, and *C*. *arcticum*).

The ANCOM-BC approach is a relatively new statistical approach to inferring differential abundances in microbiome datasets and aims to correct for bias introduced by differences in the sampling fractions across samples (Lin and Peddada, 2020), which the DeSeq2 approach does not account for. The difference in the results between the two approaches is most notable for some ASVs that had a highly negative log_2_ fold change in the DeSeq2 analysis, but did not fall below −1 in the ANCOM-BC such as *Lacibacter and Bradyrhizobium*. Interestingly, in both analyses *Pseudomonas_E* was represented by ASVs showing both significantly greater and significantly lower relative abundances for corresponding microbiological samples positive for *Listeria*. Therefore, it is possible that certain strains of *Pseudomonas_E* are putatively positively or negatively associated with *Listeria* positive samples.

### 3.3. Overall microbiome

#### 3.3.1. Abundant taxa

**Figure 7:**
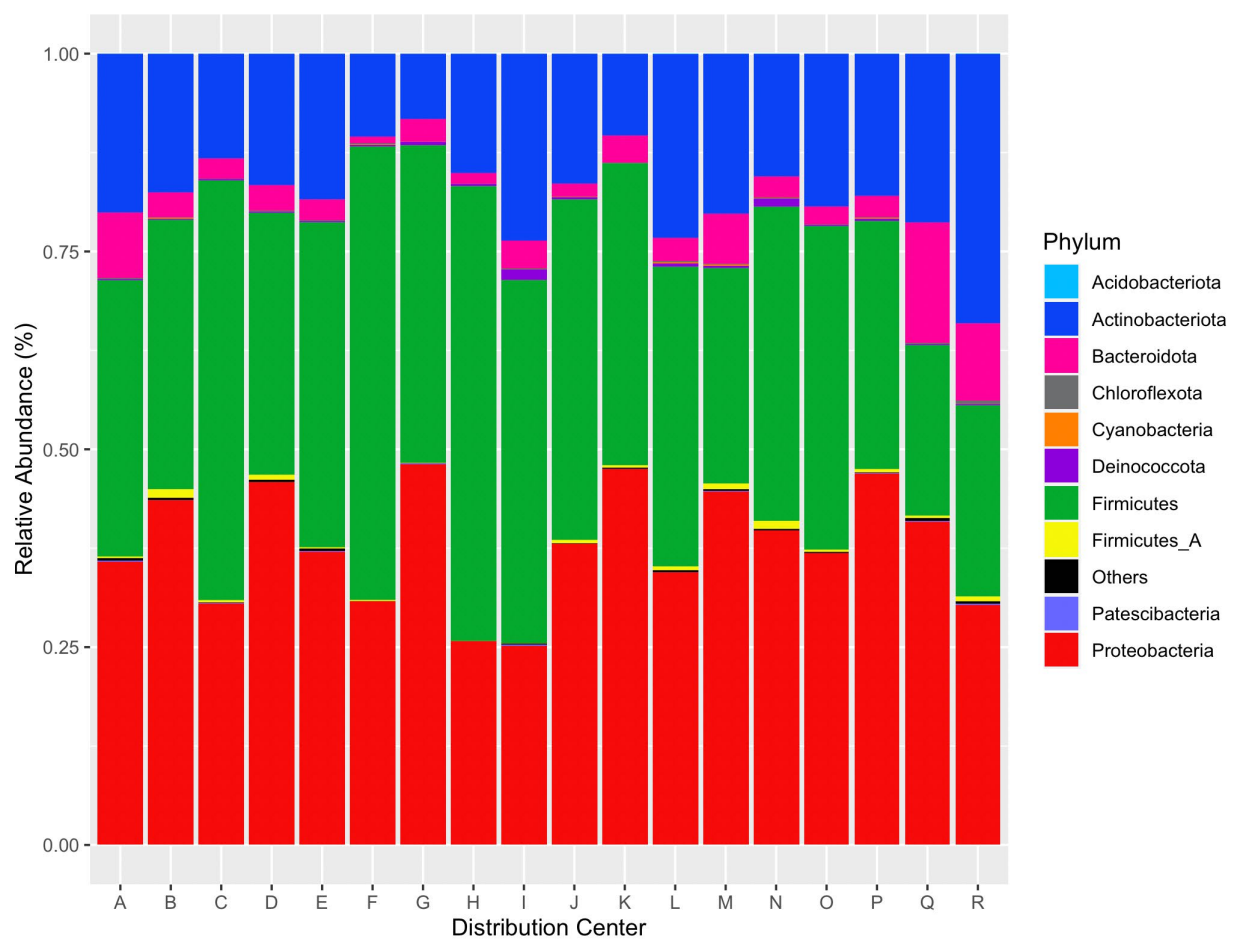
Relative abundance of the top 10 phyla per distribution center. “Others” contains the remaining identified phyla.

Abundant taxa were determined after taxonomic filtering (e.g., read removal via prevalence threshold and agglomeration). The top five phyla provided from relative abundance calculations via *phyloseq* were Proteobacteria, Firmicutes, Actinobacteriota, Bacteroidota, and Fimicutes_A. A plot of the top 10 phyla per distribution center by relative abundance is given in Figure 7.

Twelve ASVs were present in all samples. These ASVs correspond to genera *Carnobacterium_A*, *Pseudomonas_E*, *Glutamicibacter*, *Acinetobacter*, *Methylobacterium*, *Staphylococcus*, *Aerococcus*, *Sphingomonas*, *Psychrobacter*, *Kocuria*, *Citricoccus*, and *Massilia*. The top five genera across all reads were *Carnobacterium*_A (29.5% relative abundance), *Psychrobacter* (28.6%), *Pseudomonas*_*E* (14.3%), *Leaf454* (14.2%), and *Staphylococcus* (10.6%). Several species of *Carnobacterium* have been frequently isolated from food environments and foods (Leisner et al., 2007). This genus is psychrotolerant and is capable of spoiling refrigerated foods (Leisner et al., 2007; Afzal et al., 2010). As several species within this genus are tolerant to cold temperatures, it is not surprising to find this genus in microbiome data from refrigerated areas of DCs. *Psychrobacter* spp. were originally isolated from poultry (Juni and Heym, 1986) but have also been isolated from marine and Antarctic environments (Romanenko et al., 2002; Shivaji et al., 2005), and fish (Gonzalez et al., 2000). As evident by their name, *Psychrobacter* species are psychrophilic or cold-adapted and can grow at-18 to 37°C with optimum growth at 20–30°C (Yang, 2014). *Pseudomonas* is a bacteria of concern in the food industry as it can cause spoilage of fruits, vegetables, and pasteurized dairy and meat products (Gram et al., 2002; Rajmohan et al., 2002; Kumar et al., 2019) and is also cold tolerant (Meyer et al., 2004; Zhang et al., 2019). Within the GTDB, *Leaf454* corresponds to an unclassified *Aureimonas* bacteria; however, this sample is also contaminated with plant plastid DNA. Therefore, the fourth most abundant genus also represents plant DNA, which is unsurprising to find in DCs handling fresh produce. *Staphylococcus* is ubiquitous in nature and animals (Trülzsch et al., 2007). More notably, *S*. *aureus* is a foodborne bacteria and is typically linked to foods such as meats, puddings, sandwiches, and dairy products (Hennekinne et al., 2012).

#### 3.3.2. Beta diversity

**Figure 8:**
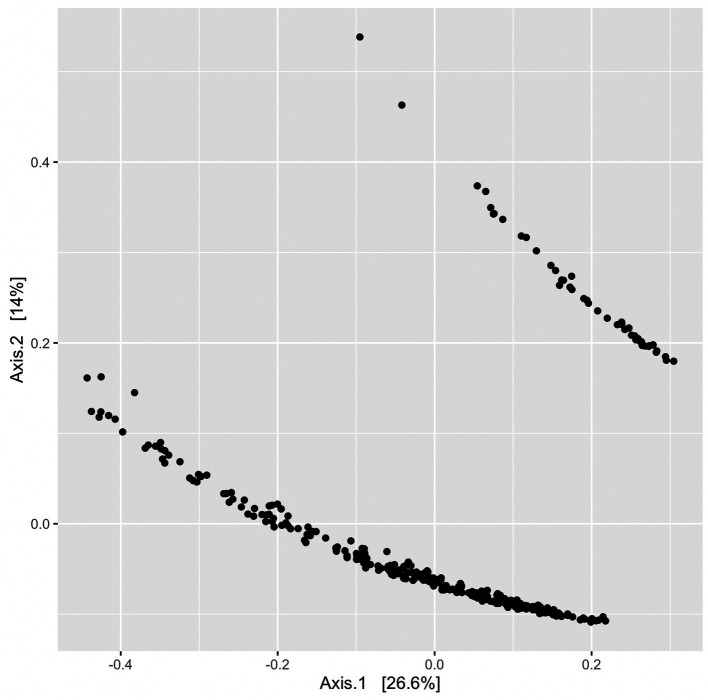
PCoA plot without groupings to illustrate clustering of ordinated UniFrac distances. Each point represents a single sample’s UniFrac distance.

Beta diversity was assessed using unweighted UniFrac distances produced from a randomly rooted maximum likelihood phylogenetic tree. Principle coordinate analysis (PCoA) plots were generated from UniFrac distances and displayed two clusters (Figure 8); however, no variables or groupings explored in this study correlated with clustering observed in the PCoA plots. Sequencing batch effects and Qubit DNA concentration ranges pre-and post-PCR were also compared and none correlated with the clustering pattern (Supplementary Figure S1 in Supplementary material).

In betadisper and PERMANOVA analyses (Table 2), most groups/variables (e.g., *Listeria*, season, dryness) demonstrated homologous dispersions or variances, but did not indicate homologous compositions. This may be further supported by the low number of common ASVs shared across DC or other variable groupings (discussed previously), which may indicate variability in microbiome composition. The only notable variable with a relatively high level of dispersion was general location (*p* = 0.002 and *F* = 4.1), indicating that variance between general sampling locations (e.g., cold storage, receiving, and cleaning areas) is significantly different. While many variables also had statistically significant *p* values from PERMANOVA analyses, they did not have large *R*^2^ statistics to indicate large variation in distances within the grouping. Grouping by season provided the largest *R*^2^ statistic (*p* = 0.001, *R*^2^ = 0.069), meaning 6.9% of the variation in distances could be explained by season. However, the number of samples taken across seasons was not equal and seasonality may also be impacted by each DC’s geographical location.

**Table 2:** Statistical tests with corresponding *p* value and *F* or *R*^2^ values from comparisons between unweighted UniFrac distances by group/variable (alpha level of 0.05).

Group/variable	Statistical test			
	Betadisper		PERMANOVA^*^	
	*p* value	*F*	*p* value	*R* ^2^
*Listeria*	0.894	0.023	0.002	0.015
DC	0.049	1.6	N/A	
General location	0.002	4.1	N/A	
Primary location	0.102	2.1	0.203	0.012
Zone	0.816	0.048	0.054	0.0062
Season	0.261	1.3	0.001	0.069
Precipitation	0.062	2.0	0.001	0.021
Geography	0.069	2.5	0.001	0.056
Dryness	0.102	2.8	0.001	0.035
Cleaning type	0.548	0.73	0.001	0.022

* Not applicable (N/A) given for PERMANOVA when assumptions are not met based on results from betadisper (rejection of null hypothesis meaning group dispersions are not homologous).

## 4. Conclusion

There was not a substantial relationship between *Listeria* abundance in 16S amplicon reads and *Listeria* presence in the microbiological samples tested. This finding suggests that 16S amplicon read-based assumptions of microbial presence within environments should be interpreted conservatively, as bacterial viability is critical for determining food safety risks. Differential abundance analysis found two taxa (*Psychrobacter* and *Pseudomonas_E*) associated with *Listeria-*positive samples. Microbiomes across individual DCs as well as within other variables (e.g., general location, geography), appeared to vary in composition, rather than dispersion, as demonstrated by betadisper and PERMANOVA analyses. The most common genera found across microbiomes were cold-tolerant species, which were unsurprising since DCs contain refrigerated areas and 71.6% (217/303) of samples were collected from these areas (e.g., cold storage, shipping/receiving docks). Additional studies are needed to examine the microbiome within food-related built environments, especially for identifying relationships between taxa and facility variables, and/or tracking communities through these environments, or the larger supply chain.

## Data availability statement

The data presented in the study are deposited in the NCBI repository, accesion number PRJNA841648.

## Author contributions

LD and LS acquired funding. AT and LD collected samples. AT, CM, and LS completed sample processing. AM completed metagenomic sequencing. AT performed bioinformatic analyses with support from HB and prepared the original manuscript. CM, LS, HB, and LD revised the manuscript. All authors contributed to the article and approved the submitted version.

## Funding

Funding for this project was made possible by the U.S. Department of Agriculture’s (USDA) Agricultural Marketing Service through grant AM170100XXXXG008. Its contents are solely the responsibility of the authors and do not necessarily represent the official views of the USDA. Additional funding and support was provided by the Center for Produce Safety.

## Conflict of interest

The authors declare that the research was conducted in the absence of any commercial or financial relationships that could be construed as a potential conflict of interest.

## Publisher’s note

All claims expressed in this article are solely those of the authors and do not necessarily represent those of their affiliated organizations, or those of the publisher, the editors and the reviewers. Any product that may be evaluated in this article, or claim that may be made by its manufacturer, is not guaranteed or endorsed by the publisher.

## References

[ref1] Afzal M. I. Jacquet T. Delaunay S. Borges F. Millière J.-B. Revol-Junelles A.-M.. (2010). *Carnobacterium maltaromaticum*: identification, isolation tools, ecology and technological aspects in dairy products. Food Microbiol. 27, 573–579. doi: 10.1016/j.fm.2010.03.019 20510773

[ref2] Altschul S. F. Gish W. Miller W. Myers E. W. Lipman D. J. (1990). Basic local alignment search tool. J. Mol. Biol. 215, 403–410. doi: 10.1016/s0022-2836(05)80360-2 2231712

[ref4] Belk A. D. Frazier A. N. Fuerniss L. K. Delmore R. Belk K. Borlee B.. (2022). A pilot study: the development of a facility-associated microbiome and its association with the presence of *Listeria* spp. in one smalll meat processing facility. Microbiology Spectrum 10:e02045. doi: 10.1128/spectrum.02045-22 PMC9603805 35980043

[ref5] Bharti R. Grimm D. G. (2021). Current challenges and best-practice protocols for microbiome analysis. Brief. Bioinform. 22, 178–193. doi: 10.1093/bib/bbz155 31848574 PMC7820839

[ref6] Bodenhofer U. Bonatesta E. Horejš-Kainrath C. Hochreiter S. (2015). Msa: an R package for multiple sequence alignment. Bioinformatics 31, 3997–3999. doi: 10.1093/bioinformatics/btv494 26315911

[ref7] Callahan B. J. McMurdie P. J. Rosen M. J. Han A. W. Johnson A. J. A. Holmes S. P. (2016a). DADA2: high-resolution sample inference from Illumina amplicon data. Nat. Methods 13, 581–583. doi: 10.1038/nmeth.3869 27214047 PMC4927377

[ref8] Callahan B. J. Sankaran K. Fukuyama J. A. McMurdie P. J. Holmes S. P. (2016b). Bioconductor workflow for microbiome data analysis: from raw reads to community analyses. F1000Res. 5:1492. doi: 10.12688/f1000research.8986.2 27508062 PMC4955027

[ref9] Carleton H. (2019). 2019: PulseNet laboratories transition to whole genome sequencing [online]. Centers for Disease Control and Prevention. Available at: https://www.cdc.gov/amd/whats-new/pulsenet-transition.html (Accessed September 21, 2021).

[ref10] Carpentier B. Cerf O. (2011). Persistence of *Listeria monocytogenes* in food industry equipment and premises. Int. J. Food Microbiol. 145, 1–8. doi: 10.1016/j.ijfoodmicro.2011.01.005 21276634

[ref11] Durso L. M. Miller D. N. Wienhold B. J. (2012). Distribution and quantification of antibiotic resistant genes and bacteria across agricultural and non-agricultural metagenomes. PLoS One 7:e48325. doi: 10.1371/journal.pone.0048325 23133629 PMC3487761

[ref12] Dzieciol M. Schornsteiner E. Muhterem-Uyar M. Stessl B. Wagner M. Schmitz-Esser S. (2016). Bacterial diversity of floor drain biofilms and drain waters in a *Listeria monocytogenes* contaminated food processing environment. Int. J. Food Microbiol. 223, 33–40. doi: 10.1016/j.ijfoodmicro.2016.02.004 26881738

[ref13] Fang H. Wang H. Cai L. Yu Y. (2015). Prevalence of antibiotic resistance genes and bacterial pathogens in long-term manured greenhouse soils as revealed by metagenomic survey. Environ. Sci. Technol. 49, 1095–1104. doi: 10.1021/es504157v 25514174

[ref14] Gonzalez C. J. Santos J. A. Garcia-Lopez M.-L. Otero A. (2000). Psychrobacters and related bacteria in freshwater fish. J. Food Prot. 63, 315–321. doi: 10.4315/0362-028x-63.3.315 10716558

[ref15] Gram L. Ravn L. Rasch M. Bruhn J. B. Christensen A. B. Givskov M. (2002). Food spoilage—interactions between food spoilage bacteria. Int. J. Food Microbiol. 78, 79–97. doi: 10.1016/S0168-1605(02)00233-7 12222639

[ref17] Hennekinne J.-A. De Buyser M.-L. Dragacci S. (2012). *Staphylococcus aureus* and its food poisoning toxins: characterization and outbreak investigation. FEMS Microbiol. Rev. 36, 815–836. doi: 10.1111/j.1574-6976.2011.00311.x 22091892

[ref18] Hitchins A.D. Jinneman K. Chen Y. (2017). BAM chapter 10: detection of Listeria monocytogenes in foods and environmental samples, and enumeration of Listeria monocytogenes in foods [online]. U.S. Food and Drug Administration. Available at: https://www.fda.gov/food/laboratory-methods-food/bam-chapter-10-detection-listeria-monocytogenes-foods-and-environmental-samples-and-enumeration (Accessed September 21, 2021).

[ref19] Hoelzer K. Sauders B. D. Sanchez M. D. Olsen P. T. Pickett M. M. Mangione K. J.. (2011). Prevalence, distribution, and diversity of *Listeria monocytogenes* in retail environments, focusing on small establishments and establishments with a history of failed inspections. J. Food Prot. 74, 1083–1095. doi: 10.4315/0362-028x.jfp-10-567 21740710

[ref20] Illumina (2021). 16S metagenomic sequencing library preparation [online]. Available at: https://support.illumina.com/content/dam/illumina-support/documents/documentation/chemistry_documentation/16s/16s-metagenomic-library-prep-guide-15044223-b.pdf (Accessed September 24, 2021).

[ref21] Jagadeesan B. Gerner-Smidt P. Allard M. W. Leuillet S. Winkler A. Xiao Y.. (2019). The use of next generation sequencing for improving food safety: translation into practice. Food Microbiol. 79, 96–115. doi: 10.1016/j.fm.2018.11.005 30621881 PMC6492263

[ref22] Jarvis K. G. White J. R. Grim C. J. Ewing L. Ottesen A. R. Beaubrun J. J.-G.. (2015). Cilantro microbiome before and after nonselective pre-enrichment for *Salmonella* using 16S rRNA and metagenomic sequencing. BMC Microbiol. 15, 1–13. doi: 10.1186/s12866-015-0497-2 26264042 PMC4534111

[ref24] Juni E. Heym G. A. (1986). *Psychrobacter immobilis* gen. Nov., sp. nov.: genospecies composed of gram-negative, aerobic, oxidase-positive coccobacilli. Int. J. Syst. Evol. Microbiol. 36, 388–391. doi: 10.1099/00207713-36-3-388

[ref25] Koutsoumanis K. Allende A. Alvarez-Ordóñez A. Bolton D. Bover-Cid S. Chemaly M.. (2019). Whole genome sequencing and metagenomics for outbreak investigation, source attribution and risk assessment of food-borne microorganisms. EFSA J. 17:e05898. doi: 10.2903/j.efsa.2019.5898 32626197 PMC7008917

[ref26] Kovac J. den Bakker H. Carroll L. M. Wiedmann M. (2017). Precision food safety: a systems approach to food safety facilitated by genomics tools. TrAC Trends Anal. Chem. 96, 52–61. doi: 10.1016/j.trac.2017.06.001

[ref27] Kumar H. Franzetti L. Kaushal A. Kumar D. (2019). *Pseudomonas fluorescens*: a potential food spoiler and challenges and advances in its detection. Ann. Microbiol. 69, 873–883. doi: 10.1007/s13213-019-01501-7

[ref28] Lahti L. Shetty S. (2017). Microbiome R package [online]. Available at: https://github.com/microbiome/microbiome (Accessed March 15, 2022).

[ref29] Leisner J. J. Laursen B. G. Prévost H. Drider D. Dalgaard P. (2007). *Carnobacterium*: positive and negative effects in the environment and in foods. FEMS Microbiol. Rev. 31, 592–613. doi: 10.1111/j.1574-6976.2007.00080.x 17696886 PMC2040187

[ref30] Leonard S. R. Mammel M. K. Lacher D. W. Elkins C. A. (2015). Application of metagenomic sequencing to food safety: detection of Shiga toxin-producing *Escherichia coli* on fresh bagged spinach. Appl. Environ. Microbiol. 81, 8183–8191. doi: 10.1128/AEM.02601-15 26386062 PMC4651076

[ref31] Leonard S. R. Mammel M. K. Lacher D. W. Elkins C. A. (2016). Strain-level discrimination of Shiga toxin-producing *Escherichia coli* in spinach using metagenomic sequencing. PLoS One 11:e0167870. doi: 10.1371/journal.pone.0167870 27930729 PMC5145215

[ref32] Lim E. S. Kim J. J. Sul W. J. Kim J.-S. Kim B. Kim H.. (2021). Metagenomic analysis of microbial composition revealed cross-contamination pathway of bacteria at a foodservice facility. Front. Microbiol. 12:790. doi: 10.3389/fmicb.2021.636329 PMC8071874 33912146

[ref33] Lin H. Peddada S. D. (2020). Analysis of compositions of microbiomes with bias correction. Nat. Commun. 11:3514. doi: 10.1038/s41467-020-17041-7 32665548 PMC7360769

[ref34] Love M. I. Huber W. Anders S. (2014). Moderated estimation of fold change and dispersion for RNA-seq data with DESeq2. Genome Biol. 15, 1–21. doi: 10.1186/s13059-014-0550-8 PMC4302049 25516281

[ref35] Lozupone C. Knight R. (2005). UniFrac: a new phylogenetic method for comparing microbial communities. Appl. Environ. Microbiol. 71, 8228–8235. doi: 10.1038/s41467-020-17041-7 16332807 PMC1317376

[ref36] McMurdie P. J. Holmes S. (2013). Phyloseq: an R package for reproducible interactive analysis and graphics of microbiome census data. PLoS One 8:e61217. doi: 10.1371/journal.pone.0061217 23630581 PMC3632530

[ref37] Meyer A. Lipson D. Martin A. Schadt C. Schmidt S. (2004). Molecular and metabolic characterization of cold-tolerant alpine soil *Pseudomonas sensu stricto*. Appl. Environ. Microbiol. 70, 483–489. doi: 10.1128/AEM.70.1.483-489.2004 14711678 PMC321299

[ref38] Mughini-Gras L. Kooh P. Augustin J.-C. David J. Fravalo P. Guillier L.. (2018). Source attribution of foodborne diseases: potentialities, hurdles, and future expectations. Front. Microbiol. 9:1983. doi: 10.3389/fmicb.2018.01983 30233509 PMC6129602

[ref39] Oksanen J. Blanchet F.G. Friendly M. Kindt R. Legendre P. McGlinn D.. (2020). Vegan: Community ecology package. R package version 2.5–7. [online]. Available at: https://CRAN.R-project.org/package=vegan (Accessed March 22, 2022).

[ref41] Ottesen A. R. Gonzalez A. Bell R. Arce C. Rideout S. Allard M.. (2013). Co-enriching microflora associated with culture based methods to detect *Salmonella* from tomato phyllosphere. PLoS One 8:e73079. doi: 10.1371/journal.pone.0073079 24039862 PMC3767688

[ref43] Rajmohan S. Dodd C. Waites W. (2002). Enzymes from isolates of *Pseudomonas fluorescens* involved in food spoilage. J. Appl. Microbiol. 93, 205–213. doi: 10.1046/j.1365-2672.2002.01674.x 12147068

[ref44] Ribot E. M. Freeman M. Hise K. B. Gerner-Smidt P. (2019). PulseNet: entering the age of next-generation sequencing. Foodborne Pathog. Dis. 16, 451–456. doi: 10.1089/fpd.2019.2634 31241352 PMC6653803

[ref45] Romanenko L. A. Schumann P. Rohde M. Lysenko A. M. Mikhailov V. V. Stackebrandt E. (2002). *Psychrobacter submarinus* sp. nov. and *Psychrobacter marincola* sp. nov., psychrophilic halophiles from marine environments. Int. J. Syst. Evol. Microbiol. 52, 1291–1297. doi: 10.1099/00207713-52-4-1291 12148642

[ref46] Ronholm J. Nasheri N. Petronella N. Pagotto F. (2016). Navigating microbiological food safety in the era of whole-genome sequencing. Clin. Microbiol. Rev. 29, 837–857. doi: 10.1128/CMR.00056-16 27559074 PMC5010751

[ref47] Schliep K. P. (2011). Phangorn: phylogenetic analysis in R. Bioinformatics 27, 592–593. doi: 10.1093/bioinformatics/btq706 21169378 PMC3035803

[ref49] Shivaji S. Reddy G. S. Suresh K. Gupta P. Chintalapati S. Schumann P.. (2005). *Psychrobacter vallis* sp. nov. and *Psychrobacter aquaticus* sp. nov., from Antarctica. Int. J. Syst. Evol. Microbiol. 55, 757–762. doi: 10.1099/ijs.0.03030-0 15774658

[ref50] Smith S. (2022). Functions to help analyze data as phyloseq objects [online]. Available at: https://schuyler-smith.github.io/phylosmith/ (Accessed March 15, 2022).

[ref51] Stevens E. L. Carleton H. A. Beal J. Tillman G. E. Lindsey R. L. Lauer A.. (2022). The use of whole-genome sequencing by the federal interagency collaboration for genomics for food and feed safety in the United States. J. Food Prot. 85, 755–772. doi: 10.4315/jfp-21-437 35259246

[ref52] Tan X. Chung T. Chen Y. Macarisin D. LaBorde L. Kovac J. (2019). The occurrence of *Listeria monocytogenes* is associated with built environment microbiota in three tree fruit processing facilities. Microbiome 7, 1–18. doi: 10.1186/s40168-019-0726-2 31431193 PMC6702733

[ref53] Tolar B. Joseph L. A. Schroeder M. N. Stroika S. Ribot E. M. Hise K. B.. (2019). An overview of PulseNet USA databases. Foodborne Pathog. Dis. 16, 457–462. doi: 10.1089/fpd.2019.2637 31066584 PMC6653802

[ref54] Townsend A. Strawn L. K. Chapman B. J. Dunn L. L. (2021). A systematic review of *Listeria* species and *Listeria monocytogenes* prevalence, persistence, and diversity throughout the fresh produce supply chain. Foods 10:1427. 34202947 10.3390/foods10061427 PMC8234284

[ref55] Townsend A. Strawn L. K. Chapman B. J. Yavelak M. Mishra A. Dunn L. L. (2022). Factors that predict *Listeria* prevalence in distribution centers handling fresh produce. Food Microbiol. 107:104065. doi: 10.1016/j.fm.2022.104065 35953185

[ref56] Trülzsch K. Grabein B. Schumann P. Mellmann A. Antonenka U. Heesemann J.. (2007). *Staphylococcus pettenkoferi* sp. nov., a novel coagulase-negative staphylococcal species isolated from human clinical specimens. Int. J. Syst. Evol. Microbiol. 57, 1543–1548. doi: 10.1099/ijs.0.64381-0 17625191

[ref57] Wickham H. (2016). ggplot2: Elegant Graphics for Data Analysis. New York: Springer-Verlag

[ref58] Yang X. (2014). “Moraxellaceae” in Encyclopedia of Food Microbiology. eds. Batt C. A. Tortorello M. L.. 2nd Edn. (Oxford: Academic Press), 826–833.

[ref59] Yang X. Noyes N. R. Doster E. Martin J. N. Linke L. M. Magnuson R. J.. (2016). Use of metagenomic shotgun sequencing technology to detect foodborne pathogens within the microbiome of the beef production chain. Appl. Environ. Microbiol. 82, 2433–2443. doi: 10.1128/AEM.00078-16 26873315 PMC4959480

[ref60] Zhang Y. Wei J. Yuan Y. Yue T. (2019). Diversity and characterization of spoilage-associated psychrotrophs in food in cold chain. Int. J. Food Microbiol. 290, 86–95. doi: 10.1016/j.ijfoodmicro.2018.09.026 30317110

