# Supplementary material for: 16S microbiome analysis of microbial communities in distribution centers handling fresh produce
Source: Front Microbiol. 2023 Jul 12;14:1041936. doi: 10.3389/fmicb.2023.1041936 (PMC10369000; doi:10.3389/fmicb.2023.1041936)
Supplement: Supplementary file 1 [file Data_Sheet_1.docx]

Supplementary Material

# Supplementary Tables

**Table S1.** List of microbiome samples (n = 303) and their characteristics. The last column provides whether the corresponding microbiological samples tested positive or negative for *Listeria.*

| **DC** | **General location** | **Primary location** | **State** | **Geography** | **Season** | **Zone** | **Days since last precip.** | **Dryness** | **Cleaning type** | **Listeria** |
| --- | --- | --- | --- | --- | --- | --- | --- | --- | --- | --- |
| A | Receiving | Floor | Michigan | Midwest | Winter | 3 | 3 | Dry | Both | Negative |
| A | Cold Storage | Floor | Michigan | Midwest | Winter | 3 | 3 | Dry | Both | Negative |
| A | Cold Storage | Floor | Michigan | Midwest | Winter | 3 | 3 | Dry | Both | Negative |
| A | Shipping | Cleaning | Michigan | Midwest | Winter | 3 | 3 | Dry | None | Negative |
| A | Shipping | Floor | Michigan | Midwest | Winter | 3 | 3 | Dry | Wet | Negative |
| A | Shipping | Floor | Michigan | Midwest | Winter | 3 | 3 | Dry | Both | Negative |
| A | Shipping | Cleaning | Michigan | Midwest | Winter | 3 | 3 | Dry | None | Negative |
| B | Receiving | Floor | Michigan | Midwest | Winter | 3 | 4 | Dry | Both | Negative |
| B | Receiving | Floor | Michigan | Midwest | Winter | 3 | 4 | Dry | Both | Negative |
| B | Receiving | Floor | Michigan | Midwest | Winter | 3 | 4 | Dry | Both | Negative |
| B | Receiving | Floor | Michigan | Midwest | Winter | 3 | 4 | Dry | Both | Negative |
| B | Cold Storage | Floor | Michigan | Midwest | Winter | 3 | 4 | Dry | Both | Negative |
| B | Shipping | Floor | Michigan | Midwest | Winter | 3 | 4 | Dry | Both | Negative |
| B | Shipping | Cleaning | Michigan | Midwest | Winter | 3 | 4 | Dry | Wet | Negative |
| C | Receiving | Floor | North Carolina | South | Winter | 3 | 4 | Dry | Both | Negative |
| C | Receiving | Floor | North Carolina | South | Winter | 3 | 4 | Dry | Both | Negative |
| C | Receiving | Cleaning | North Carolina | South | Winter | 3 | 4 | Dry | Wet | Negative |
| C | Receiving | Barrier | North Carolina | South | Winter | 3 | 4 | Dry | Wet | Negative |
| C | Receiving | Cleaning | North Carolina | South | Winter | 3 | 4 | Dry | None | Positive |
| C | Receiving | Cleaning | North Carolina | South | Winter | 3 | 4 | Wet | Wet | Negative |
| C | Receiving | Floor | North Carolina | South | Winter | 3 | 4 | Dry | Both | Negative |
| C | Cold Storage | Cleaning | North Carolina | South | Winter | 3 | 4 | Dry | None | Negative |
| C | Cold Storage | Floor | North Carolina | South | Winter | 3 | 4 | Dry | Both | Negative |
| D | Receiving | Floor | Kentucky | Midwest | Winter | 3 | 4 | Dry | Wet | Negative |
| D | Receiving | Cleaning | Kentucky | Midwest | Winter | 3 | 4 | Dry | Wet | Negative |
| D | Receiving | Floor | Kentucky | Midwest | Winter | 3 | 4 | Dry | Both | Negative |
| D | 50F Room | Floor | Kentucky | Midwest | Winter | 3 | 4 | Dry | Both | Negative |
| D | Cold Storage | Floor | Kentucky | Midwest | Winter | 3 | 4 | Dry | Dry | Negative |
| E | Cleaning Area | Cleaning | Texas | South | Winter | 3 | 0 | Dry | Wet | Negative |
| E | Receiving | Floor | Texas | South | Winter | 3 | 0 | Dry | Both | Negative |
| E | Receiving | Floor | Texas | South | Winter | 3 | 0 | Dry | Both | Negative |
| E | Receiving | Floor | Texas | South | Winter | 3 | 0 | Dry | Wet | Negative |
| E | Cold Storage | Barrier | Texas | South | Winter | 3 | 0 | Dry | Wet | Negative |
| E | 50F Room | Floor | Texas | South | Winter | 3 | 0 | Dry | Wet | Negative |
| E | 50F Room | Cleaning | Texas | South | Winter | 3 | 0 | Dry | Wet | Negative |
| F | Shipping/Receiving | Floor | Texas | South | Winter | 3 | 6 | Dry | Both | Positive |
| F | Cold Storage | Floor | Texas | South | Winter | 3 | 6 | Dry | Both | Positive |
| F | Cold Storage | Cleaning | Texas | South | Winter | 3 | 6 | Wet | Wet | Negative |
| F | Shipping/Receiving | Floor | Texas | South | Winter | 3 | 6 | Dry | Both | Negative |
| F | Shipping/Receiving | Cleaning | Texas | South | Winter | 3 | 6 | Dry | Wet | Negative |
| F | Shipping/Receiving | Barrier | Texas | South | Winter | 3 | 6 | Dry | Wet | Negative |
| G | Receiving | Barrier | Georgia | South | Fall | 3 | 0 | Dry | Wet | Negative |
| G | Receiving | Cleaning | Georgia | South | Fall | 3 | 0 | Dry | None | Positive |
| G | Receiving | Barrier | Georgia | South | Fall | 3 | 0 | Dry | Wet | Negative |
| G | Receiving | Cleaning | Georgia | South | Fall | 3 | 0 | Dry | Wet | Positive |
| G | Receiving | Floor | Georgia | South | Fall | 3 | 0 | Dry | Both | Negative |
| G | Cold Storage | Floor | Georgia | South | Fall | 3 | 0 | Dry | Dry | Negative |
| G | Cold Storage | Cleaning | Georgia | South | Fall | 3 | 0 | Dry | None | Negative |
| G | 50F Room | Floor | Georgia | South | Fall | 3 | 0 | Dry | Dry | Negative |
| G | Merge | Floor | Georgia | South | Fall | 3 | 0 | Dry | Wet | Positive |
| G | Shipping | Barrier | Georgia | South | Fall | 3 | 0 | Dry | Wet | Positive |
| G | Shipping | Cleaning | Georgia | South | Fall | 3 | 0 | Dry | Wet | Positive |
| G | Shipping | Floor | Georgia | South | Fall | 3 | 0 | Dry | Both | Positive |
| H | Shipping/Receiving | Floor | Florida | South | Fall | 3 | 0 | Dry | Both | Positive |
| H | Shipping/Receiving | Floor | Florida | South | Fall | 3 | 0 | Dry | Both | Negative |
| H | Shipping/Receiving | Floor | Florida | South | Fall | 3 | 0 | Wet | Both | Negative |
| H | Shipping/Receiving | Floor | Florida | South | Fall | 3 | 0 | Dry | Both | Negative |
| H | Shipping/Receiving | Wall | Florida | South | Fall | 3 | 0 | Dry | Wet | Negative |
| H | Shipping/Receiving | Floor | Florida | South | Fall | 3 | 0 | Dry | Both | Negative |
| H | Shipping/Receiving | Floor | Florida | South | Fall | 3 | 0 | Wet | Both | Negative |
| H | 50F Room | Floor | Florida | South | Fall | 3 | 0 | Dry | Both | Negative |
| H | 50F Room | Cleaning | Florida | South | Fall | 3 | 0 | Dry | None | Negative |
| H | 50F Room | Floor | Florida | South | Fall | 3 | 0 | Dry | Both | Negative |
| H | 50F Room | Cleaning | Florida | South | Fall | 3 | 0 | Dry | Wet | Negative |
| H | 50F Room | Barrier | Florida | South | Fall | 3 | 0 | Dry | Wet | Negative |
| H | Cold Storage | Barrier | Florida | South | Fall | 3 | 0 | Dry | Wet | Negative |
| H | Cold Storage | Floor | Florida | South | Fall | 3 | 0 | Wet | Both | Negative |
| H | Cleaning Area | Floor | Florida | South | Fall | 4 | 0 | Wet | Both | Negative |
| I | Shipping/Receiving | Floor | Florida | South | Fall | 3 | 0 | Wet | Both | Negative |
| I | Shipping/Receiving | Barrier | Florida | South | Fall | 3 | 0 | Dry | Wet | Negative |
| I | Shipping/Receiving | Floor | Florida | South | Fall | 3 | 0 | Dry | Both | Negative |
| I | Shipping/Receiving | Barrier | Florida | South | Fall | 3 | 0 | Dry | Wet | Negative |
| I | Shipping/Receiving | Floor | Florida | South | Fall | 3 | 0 | Dry | Both | Negative |
| I | Shipping/Receiving | Floor | Florida | South | Fall | 3 | 0 | Dry | Both | Positive |
| I | Shipping/Receiving | Barrier | Florida | South | Fall | 3 | 0 | Dry | Wet | Positive |
| I | Cold Storage | Barrier | Florida | South | Fall | 3 | 0 | Dry | Wet | Negative |
| I | 50F Room | Floor | Florida | South | Fall | 3 | 0 | Dry | Both | Positive |
| J | 50F Room | Floor | Pennsylvania | Northeast | Fall | 3 | 0 | Dry | Dry | Negative |
| J | 50F Room | Barrier | Pennsylvania | Northeast | Fall | 3 | 0 | Dry | Wet | Negative |
| J | Receiving | Floor | Pennsylvania | Northeast | Fall | 3 | 0 | Dry | Both | Negative |
| J | Receiving | Floor | Pennsylvania | Northeast | Fall | 3 | 0 | Wet | Both | Negative |
| J | Cold Storage | Floor | Pennsylvania | Northeast | Fall | 3 | 0 | Dry | Dry | Negative |
| J | Cold Storage | Floor | Pennsylvania | Northeast | Fall | 3 | 0 | Dry | Dry | Negative |
| J | Merge | Floor | Pennsylvania | Northeast | Fall | 3 | 0 | Dry | Dry | Negative |
| J | Shipping | Floor | Pennsylvania | Northeast | Fall | 3 | 0 | Wet | Both | Negative |
| J | Shipping | Wall | Pennsylvania | Northeast | Fall | 3 | 0 | Dry | None | Negative |
| K | Receiving | Barrier | Ohio | Midwest | Fall | 3 | 4 | Dry | Wet | Negative |
| K | Receiving | Barrier | Ohio | Midwest | Fall | 3 | 4 | Dry | Wet | Negative |
| K | Receiving | Floor | Ohio | Midwest | Fall | 3 | 4 | Dry | Dry | Negative |
| K | Receiving | Floor | Ohio | Midwest | Fall | 3 | 4 | Wet | Both | Negative |
| K | Receiving | Barrier | Ohio | Midwest | Fall | 3 | 4 | Dry | Wet | Negative |
| K | Receiving | Floor | Ohio | Midwest | Fall | 3 | 4 | Dry | Wet | Negative |
| K | Cold Storage | Floor | Ohio | Midwest | Fall | 3 | 4 | Dry | Dry | Negative |
| K | 50F Room | Barrier | Ohio | Midwest | Fall | 3 | 4 | Dry | Wet | Negative |
| K | 50F Room | Floor | Ohio | Midwest | Fall | 3 | 4 | Wet | Both | Negative |
| L | Receiving | Floor | Michigan | Midwest | Winter | 3 | 14 | Dry | Both | Negative |
| L | Receiving | Cleaning | Michigan | Midwest | Winter | 3 | 14 | Dry | Wet | Negative |
| L | Receiving | Cleaning | Michigan | Midwest | Winter | 3 | 14 | Dry | Wet | Negative |
| L | Receiving | Barrier | Michigan | Midwest | Winter | 3 | 14 | Dry | Wet | Negative |
| L | Receiving | Floor | Michigan | Midwest | Winter | 3 | 14 | Dry | Both | Negative |
| L | Receiving | Barrier | Michigan | Midwest | Winter | 3 | 14 | Dry | Wet | Negative |
| L | Receiving | Wall | Michigan | Midwest | Winter | 3 | 14 | Dry | None | Negative |
| L | Receiving | Cleaning | Michigan | Midwest | Winter | 3 | 14 | Dry | None | Negative |
| L | Receiving | Cleaning | Michigan | Midwest | Winter | 3 | 14 | Dry | None | Negative |
| L | Receiving | Floor | Michigan | Midwest | Winter | 3 | 14 | Dry | Both | Negative |
| L | 50F Room | Floor | Michigan | Midwest | Winter | 3 | 14 | Dry | Both | Negative |
| L | 50F Room | Cleaning | Michigan | Midwest | Winter | 3 | 14 | Dry | None | Negative |
| L | 50F Room | Cleaning | Michigan | Midwest | Winter | 3 | 14 | Dry | None | Negative |
| L | 50F Room | Floor | Michigan | Midwest | Winter | 3 | 14 | Dry | Both | Negative |
| L | 50F Room | Floor | Michigan | Midwest | Winter | 3 | 14 | Dry | Both | Negative |
| L | 50F Room | Barrier | Michigan | Midwest | Winter | 3 | 14 | Dry | Wet | Negative |
| L | 50F Room | Cleaning | Michigan | Midwest | Winter | 3 | 14 | Dry | None | Negative |
| L | 50F Room | Floor | Michigan | Midwest | Winter | 3 | 14 | Dry | Both | Negative |
| L | 50F Room | Floor | Michigan | Midwest | Winter | 3 | 14 | Dry | Both | Negative |
| L | Shipping | Cleaning | Michigan | Midwest | Winter | 3 | 14 | Dry | None | Negative |
| L | Shipping | Barrier | Michigan | Midwest | Winter | 3 | 14 | Dry | Wet | Negative |
| L | Shipping | Wall | Michigan | Midwest | Winter | 3 | 14 | Dry | None | Negative |
| L | Merge | Floor | Michigan | Midwest | Winter | 3 | 14 | Dry | Dry | Negative |
| L | Merge | Floor | Michigan | Midwest | Winter | 3 | 14 | Dry | Dry | Negative |
| L | Merge | Floor | Michigan | Midwest | Winter | 3 | 14 | Dry | Both | Negative |
| L | Merge | Floor | Michigan | Midwest | Winter | 3 | 14 | Dry | Both | Negative |
| L | Cold Storage | Floor | Michigan | Midwest | Winter | 3 | 14 | Dry | Both | Negative |
| L | Cleaning Area | Cleaning | Michigan | Midwest | Winter | 3 | 14 | Wet | Wet | Negative |
| L | Cleaning Area | Cleaning | Michigan | Midwest | Winter | 3 | 14 | Dry | None | Negative |
| M | Shipping/Receiving | Floor | Michigan | Midwest | Winter | 3 | 14 | Dry | Both | Negative |
| M | Shipping/Receiving | Floor | Michigan | Midwest | Winter | 3 | 14 | Dry | Both | Negative |
| M | Shipping/Receiving | Barrier | Michigan | Midwest | Winter | 3 | 14 | Dry | Wet | Negative |
| M | Shipping/Receiving | Floor | Michigan | Midwest | Winter | 3 | 14 | Dry | Both | Negative |
| M | Shipping/Receiving | Floor | Michigan | Midwest | Winter | 3 | 14 | Dry | Wet | Negative |
| M | Shipping/Receiving | Barrier | Michigan | Midwest | Winter | 3 | 14 | Dry | Wet | Negative |
| M | Shipping/Receiving | Barrier | Michigan | Midwest | Winter | 3 | 14 | Dry | Wet | Negative |
| M | Shipping/Receiving | Cleaning | Michigan | Midwest | Winter | 3 | 14 | Dry | None | Negative |
| M | Shipping/Receiving | Cleaning | Michigan | Midwest | Winter | 3 | 14 | Dry | None | Negative |
| M | Shipping/Receiving | Cleaning | Michigan | Midwest | Winter | 3 | 14 | Dry | None | Negative |
| M | Shipping/Receiving | Floor | Michigan | Midwest | Winter | 3 | 14 | Dry | Both | Negative |
| M | Shipping/Receiving | Cleaning | Michigan | Midwest | Winter | 3 | 14 | Dry | None | Negative |
| M | Shipping/Receiving | Floor | Michigan | Midwest | Winter | 3 | 14 | Dry | None | Negative |
| M | Shipping/Receiving | Wall | Michigan | Midwest | Winter | 3 | 14 | Dry | None | Negative |
| M | Shipping/Receiving | Floor | Michigan | Midwest | Winter | 3 | 14 | Dry | Both | Negative |
| M | Cold Storage | Floor | Michigan | Midwest | Winter | 3 | 14 | Dry | Both | Negative |
| M | Cold Storage | Floor | Michigan | Midwest | Winter | 3 | 14 | Wet | Both | Negative |
| M | Cold Storage | Cleaning | Michigan | Midwest | Winter | 3 | 14 | Dry | None | Negative |
| M | Cold Storage | Cleaning | Michigan | Midwest | Winter | 3 | 14 | Dry | None | Negative |
| M | Cold Storage | Floor | Michigan | Midwest | Winter | 3 | 14 | Dry | Both | Negative |
| M | Cold Storage | Floor | Michigan | Midwest | Winter | 3 | 14 | Dry | Both | Negative |
| M | Cold Storage | Cleaning | Michigan | Midwest | Winter | 3 | 14 | Dry | None | Negative |
| M | Cold Storage | Floor | Michigan | Midwest | Winter | 3 | 14 | Dry | Both | Negative |
| M | Cold Storage | Cleaning | Michigan | Midwest | Winter | 3 | 14 | Dry | None | Negative |
| M | Cold Storage | Floor | Michigan | Midwest | Winter | 3 | 14 | Dry | Both | Negative |
| M | Equipment Storage | Floor | Michigan | Midwest | Winter | 4 | 14 | Dry | Both | Negative |
| N | Cold Storage | Floor | Wisconsin | Midwest | Winter | 3 | 5 | Dry | Both | Negative |
| N | Cold Storage | Floor | Wisconsin | Midwest | Winter | 3 | 5 | Dry | Both | Negative |
| N | Cold Storage | Cleaning | Wisconsin | Midwest | Winter | 3 | 5 | Dry | Wet | Negative |
| N | Cold Storage | Floor | Wisconsin | Midwest | Winter | 3 | 5 | Dry | Both | Negative |
| N | Cold Storage | Floor | Wisconsin | Midwest | Winter | 3 | 5 | Dry | Both | Negative |
| N | Cold Storage | Floor | Wisconsin | Midwest | Winter | 3 | 5 | Dry | None | Negative |
| N | Cold Storage | Cleaning | Wisconsin | Midwest | Winter | 3 | 5 | Dry | None | Negative |
| N | Cold Storage | Barrier | Wisconsin | Midwest | Winter | 3 | 5 | Dry | Wet | Negative |
| N | Cold Storage | Floor | Wisconsin | Midwest | Winter | 3 | 5 | Dry | Both | Negative |
| N | 50F Room | Floor | Wisconsin | Midwest | Winter | 3 | 5 | Dry | Both | Negative |
| N | 50F Room | Barrier | Wisconsin | Midwest | Winter | 3 | 5 | Dry | Wet | Negative |
| N | 50F Room | Barrier | Wisconsin | Midwest | Winter | 3 | 5 | Dry | Wet | Negative |
| N | 50F Room | Floor | Wisconsin | Midwest | Winter | 3 | 5 | Dry | Both | Negative |
| N | 50F Room | Cleaning | Wisconsin | Midwest | Winter | 3 | 5 | Dry | None | Negative |
| N | 50F Room | Cleaning | Wisconsin | Midwest | Winter | 3 | 5 | Dry | None | Negative |
| N | 50F Room | Cleaning | Wisconsin | Midwest | Winter | 3 | 5 | Dry | None | Negative |
| N | 50F Room | Floor | Wisconsin | Midwest | Winter | 3 | 5 | Dry | Dry | Negative |
| N | 50F Room | Floor | Wisconsin | Midwest | Winter | 3 | 5 | Dry | Both | Negative |
| N | 50F Room | Floor | Wisconsin | Midwest | Winter | 3 | 5 | Dry | Dry | Negative |
| N | 50F Room | Floor | Wisconsin | Midwest | Winter | 3 | 5 | Dry | Both | Negative |
| N | Cold Storage | Cleaning | Wisconsin | Midwest | Winter | 3 | 5 | Wet | Wet | Negative |
| N | Cold Storage | Cleaning | Wisconsin | Midwest | Winter | 3 | 5 | Dry | None | Negative |
| N | Cold Storage | Cleaning | Wisconsin | Midwest | Winter | 3 | 5 | Dry | None | Negative |
| N | Cold Storage | Floor | Wisconsin | Midwest | Winter | 3 | 5 | Dry | Dry | Negative |
| N | Merge | Floor | Wisconsin | Midwest | Winter | 3 | 5 | Dry | Both | Negative |
| N | Merge | Floor | Wisconsin | Midwest | Winter | 3 | 5 | Dry | Wet | Negative |
| N | Receiving | Floor | Wisconsin | Midwest | Winter | 3 | 5 | Dry | Both | Negative |
| N | Receiving | Floor | Wisconsin | Midwest | Winter | 3 | 5 | Dry | Both | Negative |
| O | 50F Room | Floor | Massachusetts | Northeast | Winter | 3 | 8 | Dry | Both | Negative |
| O | 50F Room | Floor | Massachusetts | Northeast | Winter | 3 | 8 | Dry | Both | Negative |
| O | 50F Room | Floor | Massachusetts | Northeast | Winter | 3 | 8 | Dry | Both | Negative |
| O | 50F Room | Floor | Massachusetts | Northeast | Winter | 3 | 8 | Dry | Both | Negative |
| O | 50F Room | Floor | Massachusetts | Northeast | Winter | 3 | 8 | Dry | Both | Negative |
| O | 50F Room | Floor | Massachusetts | Northeast | Winter | 3 | 8 | Dry | Both | Negative |
| O | 50F Room | Cleaning | Massachusetts | Northeast | Winter | 3 | 8 | Dry | None | Negative |
| O | 50F Room | Floor | Massachusetts | Northeast | Winter | 3 | 8 | Dry | Both | Negative |
| O | Cold Storage | Floor | Massachusetts | Northeast | Winter | 3 | 8 | Dry | Both | Negative |
| O | Cold Storage | Floor | Massachusetts | Northeast | Winter | 3 | 8 | Dry | Both | Negative |
| O | Cold Storage | Floor | Massachusetts | Northeast | Winter | 3 | 8 | Dry | Both | Positive |
| O | Cold Storage | Cleaning | Massachusetts | Northeast | Winter | 3 | 8 | Dry | None | Negative |
| O | Cold Storage | Barrier | Massachusetts | Northeast | Winter | 3 | 8 | Dry | Wet | Negative |
| O | Cold Storage | Floor | Massachusetts | Northeast | Winter | 3 | 8 | Dry | Both | Negative |
| O | Cold Storage | Floor | Massachusetts | Northeast | Winter | 3 | 8 | Dry | Both | Negative |
| O | Shipping/Receiving | Floor | Massachusetts | Northeast | Winter | 3 | 8 | Dry | Wet | Negative |
| O | Shipping/Receiving | Floor | Massachusetts | Northeast | Winter | 3 | 8 | Dry | Both | Negative |
| O | Shipping/Receiving | Floor | Massachusetts | Northeast | Winter | 3 | 8 | Dry | Both | Negative |
| O | Shipping/Receiving | Cleaning | Massachusetts | Northeast | Winter | 3 | 8 | Dry | None | Negative |
| O | Shipping/Receiving | Cleaning | Massachusetts | Northeast | Winter | 3 | 8 | Dry | None | Negative |
| O | Cleaning Area | Cleaning | Massachusetts | Northeast | Winter | 3 | 8 | Dry | Wet | Negative |
| O | Cleaning Area | Cleaning | Massachusetts | Northeast | Winter | 3 | 8 | Dry | None | Negative |
| O | Cleaning Area | Cleaning | Massachusetts | Northeast | Winter | 3 | 8 | Dry | None | Negative |
| O | Equipment Storage | Cleaning | Massachusetts | Northeast | Winter | 3 | 8 | Wet | Wet | Negative |
| O | Equipment Storage | Cleaning | Massachusetts | Northeast | Winter | 3 | 8 | Dry | Wet | Negative |
| O | Equipment Storage | Cleaning | Massachusetts | Northeast | Winter | 3 | 8 | Dry | Wet | Negative |
| O | Equipment Storage | Cleaning | Massachusetts | Northeast | Winter | 3 | 8 | Dry | Wet | Negative |
| P | Cleaning Area | Floor | Maryland | Northeast | Winter | 4 | 0 | Wet | Both | Positive |
| P | Cleaning Area | Cleaning | Maryland | Northeast | Winter | 3 | 0 | Dry | Wet | Negative |
| P | Cleaning Area | Cleaning | Maryland | Northeast | Winter | 3 | 0 | Dry | None | Positive |
| P | Cleaning Area | Floor | Maryland | Northeast | Winter | 4 | 0 | Wet | Both | Negative |
| P | Cleaning Area | Cleaning | Maryland | Northeast | Winter | 3 | 0 | Dry | Wet | Negative |
| P | Cleaning Area | Cleaning | Maryland | Northeast | Winter | 3 | 0 | Dry | None | Negative |
| P | Cleaning Area | Floor | Maryland | Northeast | Winter | 4 | 0 | Dry | Both | Negative |
| P | Cleaning Area | Cleaning | Maryland | Northeast | Winter | 3 | 0 | Dry | None | Negative |
| P | Cleaning Area | Cleaning | Maryland | Northeast | Winter | 3 | 0 | Dry | None | Negative |
| P | Cleaning Area | Cleaning | Maryland | Northeast | Winter | 3 | 0 | Dry | None | Negative |
| P | Cleaning Area | Cleaning | Maryland | Northeast | Winter | 3 | 0 | Dry | None | Negative |
| P | Cleaning Area | Cleaning | Maryland | Northeast | Winter | 3 | 0 | Dry | None | Negative |
| P | Cleaning Area | Cleaning | Maryland | Northeast | Winter | 3 | 0 | Dry | None | Negative |
| P | Shipping/Receiving | Floor | Maryland | Northeast | Winter | 3 | 0 | Dry | Both | Negative |
| P | Shipping/Receiving | Floor | Maryland | Northeast | Winter | 3 | 0 | Dry | Both | Negative |
| P | Shipping/Receiving | Cleaning | Maryland | Northeast | Winter | 3 | 0 | Dry | Wet | Negative |
| P | Shipping/Receiving | Wall | Maryland | Northeast | Winter | 3 | 0 | Dry | None | Negative |
| P | Shipping/Receiving | Floor | Maryland | Northeast | Winter | 3 | 0 | Dry | Both | Negative |
| P | Shipping/Receiving | Floor | Maryland | Northeast | Winter | 3 | 0 | Dry | Both | Negative |
| P | Shipping/Receiving | Floor | Maryland | Northeast | Winter | 3 | 0 | Dry | None | Negative |
| P | Shipping/Receiving | Floor | Maryland | Northeast | Winter | 3 | 0 | Dry | Both | Negative |
| P | Shipping/Receiving | Cleaning | Maryland | Northeast | Winter | 3 | 0 | Dry | Wet | Negative |
| P | Shipping/Receiving | Floor | Maryland | Northeast | Winter | 3 | 0 | Dry | Both | Negative |
| P | Shipping/Receiving | Cleaning | Maryland | Northeast | Winter | 3 | 0 | Dry | None | Negative |
| P | Shipping/Receiving | Cleaning | Maryland | Northeast | Winter | 3 | 0 | Dry | Both | Negative |
| P | Cold Storage | Floor | Maryland | Northeast | Winter | 3 | 0 | Dry | Both | Negative |
| P | Cold Storage | Floor | Maryland | Northeast | Winter | 3 | 0 | Dry | Both | Negative |
| P | Cold Storage | Floor | Maryland | Northeast | Winter | 3 | 0 | Dry | Both | Negative |
| P | Cold Storage | Floor | Maryland | Northeast | Winter | 3 | 0 | Dry | Both | Positive |
| P | Cold Storage | Floor | Maryland | Northeast | Winter | 3 | 0 | Dry | Both | Negative |
| P | Cold Storage | Floor | Maryland | Northeast | Winter | 3 | 0 | Dry | Both | Negative |
| P | 50F Room | Floor | Maryland | Northeast | Winter | 3 | 0 | Dry | Both | Negative |
| P | 50F Room | Floor | Maryland | Northeast | Winter | 3 | 0 | Dry | Both | Negative |
| Q | Cold Storage | Floor | New York | Northeast | Spring | 3 | 3 | Wet | Both | Negative |
| Q | Cold Storage | Floor | New York | Northeast | Spring | 3 | 3 | Dry | Both | Negative |
| Q | Cold Storage | Floor | New York | Northeast | Spring | 3 | 3 | Wet | Both | Negative |
| Q | Cold Storage | Floor | New York | Northeast | Spring | 3 | 3 | Wet | Both | Negative |
| Q | Cold Storage | Floor | New York | Northeast | Spring | 3 | 3 | Wet | Both | Negative |
| Q | Cold Storage | Floor | New York | Northeast | Spring | 3 | 3 | Dry | Both | Negative |
| Q | Cold Storage | Barrier | New York | Northeast | Spring | 3 | 3 | Dry | Dry | Negative |
| Q | Cold Storage | Cleaning | New York | Northeast | Spring | 3 | 3 | Wet | Wet | Negative |
| Q | Cold Storage | Cleaning | New York | Northeast | Spring | 3 | 3 | Dry | Wet | Negative |
| Q | Cold Storage | Cleaning | New York | Northeast | Spring | 3 | 3 | Dry | None | Negative |
| Q | Cold Storage | Cleaning | New York | Northeast | Spring | 3 | 3 | Dry | None | Negative |
| Q | Cold Storage | Cleaning | New York | Northeast | Spring | 3 | 3 | Dry | None | Negative |
| Q | Cold Storage | Cleaning | New York | Northeast | Spring | 3 | 3 | Dry | Wet | Negative |
| Q | Cold Storage | Floor | New York | Northeast | Spring | 3 | 3 | Dry | Both | Positive |
| Q | Cold Storage | Floor | New York | Northeast | Spring | 3 | 3 | Wet | Both | Negative |
| Q | Cold Storage | Floor | New York | Northeast | Spring | 3 | 3 | Dry | Dry | Negative |
| Q | Cold Storage | Floor | New York | Northeast | Spring | 3 | 3 | Dry | Both | Negative |
| Q | Cold Storage | Floor | New York | Northeast | Spring | 3 | 3 | Dry | Both | Negative |
| Q | Cold Storage | Cleaning | New York | Northeast | Spring | 3 | 3 | Dry | Wet | Negative |
| Q | Cold Storage | Cleaning | New York | Northeast | Spring | 3 | 3 | Dry | None | Negative |
| Q | Cold Storage | Floor | New York | Northeast | Spring | 3 | 3 | Dry | Both | Negative |
| Q | Cold Storage | Barrier | New York | Northeast | Spring | 3 | 3 | Dry | Dry | Negative |
| Q | Shipping/Receiving | Floor | New York | Northeast | Spring | 3 | 3 | Dry | Both | Negative |
| Q | 50F Room | Floor | New York | Northeast | Spring | 3 | 3 | Dry | Both | Negative |
| Q | 50F Room | Floor | New York | Northeast | Spring | 3 | 3 | Dry | Both | Negative |
| Q | Shipping/Receiving | Barrier | New York | Northeast | Spring | 3 | 3 | Dry | Dry | Negative |
| Q | 50F Room | Floor | New York | Northeast | Spring | 3 | 3 | Dry | Both | Negative |
| Q | Banana Rooms | Floor | New York | Northeast | Spring | 3 | 3 | Dry | Both | Negative |
| Q | Banana Rooms | Floor | New York | Northeast | Spring | 3 | 3 | Dry | Both | Negative |
| Q | Banana Rooms | Floor | New York | Northeast | Spring | 3 | 3 | Wet | Both | Negative |
| Q | Equipment Storage | Cleaning | New York | Northeast | Spring | 3 | 3 | Dry | None | Negative |
| R | Cold Storage | Cleaning | Pennsylvania | Northeast | Spring | 3 | 3 | Dry | None | Negative |
| R | Cold Storage | Cleaning | Pennsylvania | Northeast | Spring | 3 | 3 | Dry | None | Negative |
| R | Cold Storage | Floor | Pennsylvania | Northeast | Spring | 3 | 3 | Wet | Both | Negative |
| R | Cold Storage | Cleaning | Pennsylvania | Northeast | Spring | 3 | 3 | Dry | Wet | Negative |
| R | Cold Storage | Floor | Pennsylvania | Northeast | Spring | 3 | 3 | Wet | Both | Negative |
| R | Cold Storage | Floor | Pennsylvania | Northeast | Spring | 3 | 3 | Wet | Both | Negative |
| R | Cold Storage | Floor | Pennsylvania | Northeast | Spring | 3 | 3 | Wet | Both | Negative |
| R | Cold Storage | Floor | Pennsylvania | Northeast | Spring | 3 | 3 | Wet | Both | Negative |
| R | Cold Storage | Barrier | Pennsylvania | Northeast | Spring | 3 | 3 | Wet | Dry | Negative |
| R | Cold Storage | Floor | Pennsylvania | Northeast | Spring | 3 | 3 | Dry | Both | Negative |
| R | Cold Storage | Floor | Pennsylvania | Northeast | Spring | 3 | 3 | Dry | Both | Negative |
| R | Cold Storage | Floor | Pennsylvania | Northeast | Spring | 3 | 3 | Dry | Both | Negative |
| R | Cold Storage | Floor | Pennsylvania | Northeast | Spring | 3 | 3 | Dry | Both | Negative |
| R | Cold Storage | Cleaning | Pennsylvania | Northeast | Spring | 3 | 3 | Dry | None | Negative |
| R | Cold Storage | Cleaning | Pennsylvania | Northeast | Spring | 3 | 3 | Dry | None | Negative |
| R | Cold Storage | Barrier | Pennsylvania | Northeast | Spring | 3 | 3 | Dry | Dry | Negative |
| R | Shipping/Receiving | Floor | Pennsylvania | Northeast | Spring | 3 | 3 | Dry | Both | Negative |
| R | Cold Storage | Floor | Pennsylvania | Northeast | Spring | 3 | 3 | Dry | Both | Negative |
| R | Cold Storage | Barrier | Pennsylvania | Northeast | Spring | 3 | 3 | Dry | Dry | Negative |
| R | Cold Storage | Barrier | Pennsylvania | Northeast | Spring | 3 | 3 | Wet | Dry | Negative |
| R | Shipping/Receiving | Floor | Pennsylvania | Northeast | Spring | 3 | 3 | Dry | Both | Negative |
| R | Shipping/Receiving | Floor | Pennsylvania | Northeast | Spring | 3 | 3 | Dry | Both | Negative |
| R | Banana Rooms | Floor | Pennsylvania | Northeast | Spring | 3 | 3 | Dry | Both | Negative |
| R | Banana Rooms | Floor | Pennsylvania | Northeast | Spring | 3 | 3 | Dry | Both | Negative |
| R | Banana Rooms | Barrier | Pennsylvania | Northeast | Spring | 3 | 3 | Dry | Dry | Negative |
| R | Banana Rooms | Floor | Pennsylvania | Northeast | Spring | 3 | 3 | Dry | Both | Negative |
| R | Cleaning Area | Floor | Pennsylvania | Northeast | Spring | 4 | 3 | Wet | Both | Negative |
| R | 50F Room | Floor | Pennsylvania | Northeast | Spring | 3 | 3 | Dry | Dry | Negative |
| R | 50F Room | Floor | Pennsylvania | Northeast | Spring | 3 | 3 | Dry | Both | Negative |
| R | 50F Room | Cleaning | Pennsylvania | Northeast | Spring | 3 | 3 | Dry | None | Negative |
| R | 50F Room | Floor | Pennsylvania | Northeast | Spring | 3 | 3 | Dry | Both | Negative |
| R | 50F Room | Floor | Pennsylvania | Northeast | Spring | 3 | 3 | Dry | Both | Negative |
| R | Cold Storage | Barrier | Pennsylvania | Northeast | Spring | 3 | 3 | Dry | Dry | Negative |
| R | Cold Storage | Barrier | Pennsylvania | Northeast | Spring | 3 | 3 | Dry | Dry | Negative |

**Table S2.** 16S amplicon read characteristics before and after taxonomic filtering

| **Read statistic** | **Stage of workflow** | | |
| --- | --- | --- | --- |
|  | **After *dada2*** | **Overall microbiome** | ***Listeria*-targeted microbiome** |
| Total number of samples | 317 | 302 | 303 |
| Total reads | 45,718,823 | 38,909,360 | 45,685,541 |
| Average reads per sample | 144,223 | 128,838 | 150,777 |
| Range of reads per sample | 23 to 1,102,817 | 10,171 to 912,264 | 10,722 to 1,102,817 |
| Median reads per sample | 125,075 | 110,028 | 128,545 |
| Total number of singletons | 7,360 | 0 | 3,617 |

**Table S3.** *Listeria*-identified ASVs within 16S amplicon reads

| ***Listeria* clade classification** | **Percent identity** | **ASV sequence** |
| --- | --- | --- |
| *Listeria sensu stricto* | 99.06 | GGAATCTTCCGCAATGGACGAAAGTCTGACGGAGCAACGCCGCGTGTATGAAGAAGGTTTTCGGATCGTAAAGTACTGTTGTTAGAGAAGAACAAGGATAAGAGTAACTGCTTGTCCCTTGACGGTATCTAACCAGAAAGCCACGGCTAACTACGTGCCAGCAGCCGCGGTAATACGTAGGTGGCAAGCGTTGTCCGGATTTATTGGGCGTAAAGCGCGCGCAGGCGGTCTTTTAAGTCTGATGTGAAAGCCCACGGCTCAACCGTGGAGGGTCATTGGAAACTGGAAGACTTGAGTGCAGAAGAGGAGAGTGGAATTCCACGTGTAGCGGTGAAATGCGTAGAGATGTGGAGGAACACCAGTGGCGAAGGCGACTCTCTGGTCTGTAACTGACGCTGAGGCGCGAAAGCGTGGGGAGCAAACAG |
| *Listeria sensu stricto* | 99.29 | GGAATCTTCCGCAATGGACGAAAGTCTGACGGAGCAACGCCGCGTGTATGAAGAAGGTTTTCGGATCGTAAAGTACTGTTGTTAGAGAAGAACAAGGATAAGAGTAACTGCTTGTCCCTTGACGGTATCTAACCAGAAAGCCACGGCTAACTACGTGCCAGCAGCCGCGGTAATACGTAGGTGGCAAGCGTTGTCCGGATTTATTGGGCGTAAAGCGCGCGCAGGCGGTCTTTTAAGTCTGATGTGAAAGCCCCCGGCTCAACCGGGGAGGGTCATTGGAAACTGGGAGACTTGAGTACAGAAGAGGAGAGTGGAATTCCACGTGTAGCGGTGAAATGCGTAGATATGTGGAGGAACACCAGTGGCGAAGGCGACTCTCTGGTCTGTAACTGACGCTGAGGCGCGAAAGCGTGGGGAGCAAACAG |
| *Listeria sensu stricto* | 100.00 | GGAATCTTCCGCAATGGACGAAAGTCTGACGGAGCAACGCCGCGTGTATGAAGAAGGTTTTCGGATCGTAAAGTACTGTTGTTAGAGAAGAACAAGGATAAGAGTAACTGCTTGTCCCTTGACGGTATCTAACCAGAAAGCCACGGCTAACTACGTGCCAGCAGCCGCGGTAATACGTAGGTGGCAAGCGTTGTCCGGATTTATTGGGCGTAAAGCGCGCGCAGGCGGTCTTTTAAGTCTGATGTGAAAGCCCCCGGCTTAACCGGGGAGGGTCATTGGAAACTGGAAGACTGGAGTGCAGAAGAGGAGAGTGGAATTCCACGTGTAGCGGTGAAATGCGTAGATATGTGGAGGAACACCAGTGGCGAAGGCGACTCTCTGGTCTGTAACTGACGCTGAGGCGCGAAAGCGTGGGGAGCAAACAG |
| *Listeria sensu lato* | 100.00 | GGAATCTTCCGCAATGGACGAAAGTCTGACGGAGCAACGCCGCGTGTGTGAAGAAGGTTTTCGGATCGTAAAGCACTGTTGTTAGAGAAGAACAAGGATAAGAGTAACTGCTTGTCCCTTGACGGTATCTAACCAGAAAGCCACGGCTAACTACGTGCCAGCAGCCGCGGTAATACGTAGGTGGCAAGCGTTGTCCGGAATTATTGGGCGTAAAGCGCGCGCAGGCGGTTTCTTAAGTCTGATGTGAAAGCCCCCGGCTTAACCGGGGAGGGTCATTGGAAACTGGGAGACTTAGAGTGCAGAAGAGGAGAGTGGAATTCCATGTGTAGCGGTGAAATGCGTAGATATATGGAGGAACACCAGTGGCGAAGGCGACTCTCTGGTCTGTAACTGACGCTGAGGCGCGAAAGCGTGGGGAGCAAACAG |
| Undetermined | 98.82 | GGAATCTTCCGCAATGGACGAAAGTCTGACGGAGCAACGCCGCGTGTGTGAAGAAGGTTTTCGGATCGTAAAGCACTGTTGTTAGAGAAGAACAAGGATAAGGTTACTACTTATCCCCTGACGGTATCTAACCAGAAAGCCACGGCTAACTACGTGCCAGCAGCCGCGGTAATACGTAGGTGGCAAGCGTTGTCCGGAATTATTGGGCGTAAAGCGCGCGCAGGCGGTTTCTTAAGTTGGGTGTGAAAGCCCTCGGCTCAACCGAGGAGGGTCACTCAAAACTGGGAGACTGGAGTGCAGAAGAGGAGAGTGGAATTCCATGTGTAGCGGTGAAATGCGTAGATATATGGAGGAACACCAGTGGCGAAGGCGGCTCTCTGGTCTGTAACTGACGCTGAGGCGCGAAAGCGTGGGGAGCAAACAG |

**Table S4.** List of phyla, families, and genera along with their corresponding adjusted p-value and log2 fold change after differential abundance analysis for microbiological samples positive and negative for *Listeria*

| **Phylum** | **Family** | **Genus** | **Log2 fold change** |
| --- | --- | --- | --- |
| *Proteobacteria* | *Moraxellaceae* | *Psychrobacter* | 4.16 |
| *Proteobacteria* | *Pseudomonadaceae* | *Pseudomonas_E* | 2.84 |
| *Actinobacteriota* | *Micrococcaceae* | *Arthrobacter_A* | -2.00 |
| *Actinobacteriota* | *Microbacteriaceae* | *Rathayibacter* | -2.19 |
| *Actinobacteriota* | *Micrococcaceae* | *Citricoccus* | -2.20 |
| *Proteobacteria* | *Moraxellaceae* | *Acinetobacter* | -2.24 |
| *Firmicutes* | *Bacillaceae_H* | *Bacillus_C* | -2.32 |
| *Proteobacteria* | *Sphingomonadaceae* | *K2R01-6* | -2.42 |
| *Actinobacteriota* | *Micrococcaceae* | *Arthrobacter_I* | -2.44 |
| *Proteobacteria* | *Sphingomonadaceae* | *Novosphingobium* | -2.49 |
| *Firmicutes* | *Planococcaceae* | *Planococcus* | -2.51 |
| *Actinobacteriota* | *Microbacteriaceae* | *Unclassified* | -2.55 |
| *Actinobacteriota* | *Mycobacteriaceae* | *Rhodococcus* | -2.67 |
| *Actinobacteriota* | *Microbacteriaceae* | *Unclassified* | -2.67 |
| *Actinobacteriota* | *Streptomycetaceae* | *Streptomyces* | -2.71 |
| *Actinobacteriota* | *Micrococcaceae* | *Arthrobacter_F* | -2.71 |
| *Actinobacteriota* | *Micrococcaceae* | *Arthrobacter_I* | -2.71 |
| *Proteobacteria* | *Rhodobacteraceae* | *Defluviimonas_B* | -2.73 |
| *Proteobacteria* | *Beijerinckiaceae* | *Methylobacterium* | -2.74 |
| *Proteobacteria* | *Sphingomonadaceae* | *Sphingomonas* | -2.76 |
| *Proteobacteria* | *Pseudomonadaceae* | *Pseudomonas_E* | -2.79 |
| *Actinobacteriota* | *Propionibacteriaceae* | *Microlunatus* | -2.79 |
| *Firmicutes* | *Aerococcaceae* | *Jeotgalibaca* | -2.82 |
| *Firmicutes* | *Lactobacillaceae* | *Leuconostoc* | -2.87 |
| *Proteobacteria* | *Pseudomonadaceae* | *Pseudomonas_E* | -2.92 |
| *Proteobacteria* | *Burkholderiaceae* | *Janthinobacterium* | -2.94 |
| *Actinobacteriota* | *Microbacteriaceae* | *Schumannella* | -2.95 |
| *Firmicutes* | *Enterococcaceae* | *Enterococcus* | -3.02 |
| *Proteobacteria* | *Enterobacteriaceae* | *Pseudescherichia* | -3.03 |
| *Proteobacteria* | *Sphingomonadaceae* | *K2R01-6* | -3.04 |
| *Actinobacteriota* | *Micrococcaceae* | *Arthrobacter_A* | -3.07 |
| *Proteobacteria* | *Acetobacteraceae* | *Paracraurococcus* | -3.07 |
| *Actinobacteriota* | *Nocardioidaceae* | *Nocardioides* | -3.08 |
| *Proteobacteria* | *Pseudomonadaceae* | *Pseudomonas_E* | -3.10 |
| *Firmicutes* | *Staphylococcaceae* | *Staphylococcus* | -3.10 |
| *Proteobacteria* | *Beijerinckiaceae* | *Methylobacterium* | -3.13 |
| *Proteobacteria* | *Burkholderiaceae* | *Massilia* | -3.15 |
| *Actinobacteriota* | *Mycobacteriaceae* | *Corynebacterium* | -3.15 |
| *Actinobacteriota* | *Microbacteriaceae* | *Root112D2* | -3.16 |
| *Proteobacteria* | *Devosiaceae* | *Devosia* | -3.16 |
| *Actinobacteriota* | *Microbacteriaceae* | *Compostimonas* | -3.17 |
| *Proteobacteria* | *Burkholderiaceae* | *Albidiferax* | -3.18 |
| *Proteobacteria* | *Pseudomonadaceae* | *Pseudomonas_E* | -3.19 |
| *Proteobacteria* | *Burkholderiaceae* | *Massilia* | -3.21 |
| *Actinobacteriota* | *Micrococcaceae* | *Nesterenkonia* | -3.24 |
| *Proteobacteria* | *Enterobacteriaceae* | *Enterobacter_D* | -3.26 |
| *Actinobacteriota* | *Micrococcaceae* | *Arthrobacter_A* | -3.29 |
| *Proteobacteria* | *Pseudomonadaceae* | *Pseudomonas_E* | -3.33 |
| *Bacteroidota* | *Flavobacteriaceae* | *Flavobacterium* | -3.34 |
| *Bacteroidota* | *Sphingobacteriaceae* | *Pedobacter* | -3.34 |
| *Firmicutes* | *Streptococcaceae* | *Streptococcus* | -3.34 |
| *Bacteroidota* | *Spirosomaceae* | *Dyadobacter* | -3.35 |
| *Proteobacteria* | *Burkholderiaceae* | *Massilia* | -3.37 |
| *Bacteroidota* | *Flavobacteriaceae* | *Flavobacterium* | -3.37 |
| *Actinobacteriota* | *Propionibacteriaceae* | *Cutibacterium* | -3.38 |
| *Proteobacteria* | *Rhodobacteraceae* | *Defluviimonas_B* | -3.42 |
| *Proteobacteria* | *Sphingomonadaceae* | *Sphingomonas_A* | -3.42 |
| *Firmicutes* | *Planococcaceae* | *Planococcus* | -3.44 |
| *Proteobacteria* | *Enterobacteriaceae* | *Erwinia* | -3.44 |
| *Proteobacteria* | *Pseudomonadaceae* | *Pseudomonas_E* | -3.44 |
| *Bacteroidota* | *Sphingobacteriaceae* | *Pedobacter* | -3.45 |
| *Proteobacteria* | *Burkholderiaceae* | *Variovorax* | -3.46 |
| *Proteobacteria* | *Sphingomonadaceae* | *Elkelangia* | -3.47 |
| *Deinococcota* | *Deinococcaceae* | *Deinococcus* | -3.48 |
| *Proteobacteria* | *Pseudomonadaceae* | *Pseudomonas_A* | -3.48 |
| *Proteobacteria* | *Rhizobiaceae* | *Neorhizobium* | -3.51 |
| *Actinobacteriota* | *Propionibacteriaceae* | *Friedmanniella* | -3.52 |
| *Bacteroidota* | *Flavobacteriaceae* | *Flavobacterium* | -3.54 |
| *Proteobacteria* | *Burkholderiaceae* | *Unclassified* | -3.55 |
| *Proteobacteria* | *Burkholderiaceae* | *Massilia* | -3.55 |
| *Proteobacteria* | *Burkholderiaceae* | *Massilia* | -3.55 |
| *Actinobacteriota* | *Propionibacteriaceae* | *Friedmanniella* | -3.55 |
| *Firmicutes_A* | *Peptostreptococcaceae* | *Romboutsia_A* | -3.56 |
| *Proteobacteria* | *Burkholderiaceae* | *Massilia* | -3.57 |
| *Proteobacteria* | *Pseudomonadaceae* | *Pseudomonas_E* | -3.58 |
| *Actinobacteriota* | *Propionibacteriaceae* | *Luteococcus* | -3.59 |
| *Firmicutes* | *Planococcaceae* | *Planococcus* | -3.61 |
| *Bacteroidota* | *Flavobacteriaceae* | *Flavobacterium* | -3.62 |
| *Actinobacteriota* | *Nocardioidaceae* | *Aeromicrobium* | -3.62 |
| *Bacteroidota* | *Flavobacteriaceae* | *Flavobacterium* | -3.64 |
| *Bacteroidota* | *Sphingobacteriaceae* | *Pedobacter* | -3.64 |
| *Proteobacteria* | *Enterobacteriaceae* | *Pectobacterium* | -3.64 |
| *Proteobacteria* | *Burkholderiaceae* | *Massilia* | -3.70 |
| *Actinobacteriota* | *Micrococcaceae* | *Citricoccus* | -3.70 |
| *Proteobacteria* | *Pseudomonadaceae* | *Pseudomonas_A* | -3.73 |
| *Bacteroidota* | *Sphingobacteriaceae* | *Pedobacter* | -3.73 |
| *Actinobacteriota* | *Micrococcaceae* | *Arthrobacter_A* | -3.74 |
| *Proteobacteria* | *Rhizobiaceae* | *Mycoplana* | -3.75 |
| *Proteobacteria* | *Acetobacteraceae* | *Roseomonas* | -3.76 |
| *Actinobacteriota* | *Micrococcaceae* | *Unclassified* | -3.76 |
| *Proteobacteria* | *Burkholderiaceae* | *Massilia* | -3.77 |
| *Bacteroidota* | *Sphingobacteriaceae* | *Sphingobacterium* | -3.78 |
| *Firmicutes* | *Lactobacillaceae* | *Ligilactobacillus* | -3.78 |
| *Proteobacteria* | *Sphingomonadaceae* | *B12* | -3.79 |
| *Proteobacteria* | *Rhodanobacteraceae* | *Luteibacter* | -3.80 |
| *Proteobacteria* | *Burkholderiaceae* | *Massilia* | -3.81 |
| *Actinobacteriota* | *Microbacteriaceae* | *Unclassified* | -3.83 |
| *Proteobacteria* | *Moraxellaceae* | *Acinetobacter* | -3.83 |
| *Actinobacteriota* | *Geodermatophilaceae* | *Modestobacter* | -3.85 |
| *Proteobacteria* | *Rhizobiaceae* | *Neorhizobium* | -3.86 |
| *Proteobacteria* | *Kaistiaceae* | *Kaistia* | -3.87 |
| *Actinobacteriota* | *Micrococcaceae* | *Arthrobacter_A* | -3.89 |
| *Proteobacteria* | *Enterobacteriaceae* | *Pectobacterium* | -3.89 |
| *Firmicutes* | *Carnobacteriaceae* | *Carnobacterium_A* | -3.90 |
| *Proteobacteria* | *Burkholderiaceae* | *Massilia* | -3.91 |
| *Actinobacteriota* | *Micrococcaceae* | *Citricoccus* | -3.91 |
| *Firmicutes* | *Bacillaceae_A* | *Bacillus_AC* | -3.93 |
| *Actinobacteriota* | *Microbacteriaceae* | *Naasia* | -3.93 |
| *Actinobacteriota* | *Nocardioidaceae* | *Aeromicrobium* | -3.94 |
| *Actinobacteriota* | *Micrococcaceae* | *Unclassified* | -3.96 |
| *Myxococcota* | *Myxococcaceae* | *Corallococcus* | -3.97 |
| *Firmicutes* | *Planococcaceae* | *Planococcus* | -3.98 |
| *Actinobacteriota* | *Mycobacteriaceae* | *Williamsia* | -3.98 |
| *Proteobacteria* | *Burkholderiaceae* | *Massilia* | -3.99 |
| *Actinobacteriota* | *Microbacteriaceae* | *Unclassified* | -4.01 |
| *Proteobacteria* | *Devosiaceae* | *Devosia* | -4.02 |
| *Proteobacteria* | *Burkholderiaceae* | *Variovorax* | -4.02 |
| *Proteobacteria* | *Sphingomonadaceae* | *Unclassified* | -4.02 |
| *Actinobacteriota* | *Nocardioidaceae* | *Nocardioides* | -4.02 |
| *Proteobacteria* | *Pseudomonadaceae* | *Pseudomonas_E* | -4.03 |
| *Bacteroidota* | *Weeksellaceae* | *Kaistella* | -4.05 |
| *Actinobacteriota* | *Micrococcaceae* | *Unclassified* | -4.05 |
| *Actinobacteriota* | *Mycobacteriaceae* | *Corynebacterium* | -4.10 |
| *Proteobacteria* | *Burkholderiaceae* | *Massilia* | -4.10 |
| *Proteobacteria* | *Pseudomonadaceae* | *Pseudomonas_E* | -4.11 |
| *Proteobacteria* | *Rhizobiaceae* | *Leaf454* | -4.12 |
| *Bacteroidota* | *Weeksellaceae* | *Kaistella* | -4.14 |
| *Firmicutes* | *Aerococcaceae* | *Facklamia_A* | -4.14 |
| *Proteobacteria* | *Enterobacteriaceae* | *Erwinia_B* | -4.15 |
| *Bacteroidota* | *Flavobacteriaceae* | *Flavobacterium* | -4.15 |
| *Firmicutes* | *Streptococcaceae* | *Lactococcus* | -4.16 |
| *Proteobacteria* | *Rhodobacteraceae* | *Defluviimonas_B* | -4.20 |
| *Proteobacteria* | *Sphingomonadaceae* | *K2R01-6* | -4.24 |
| *Actinobacteriota* | *Streptomycetaceae* | *Streptomyces* | -4.26 |
| *Actinobacteriota* | *Microbacteriaceae* | *Compostimonas* | -4.26 |
| *Actinobacteriota* | *Mycobacteriaceae* | *Rhodococcus* | -4.26 |
| *Proteobacteria* | *Enterobacteriaceae* | *Pectobacterium* | -4.26 |
| *Proteobacteria* | *Sphingomonadaceae* | *Sphingomonas* | -4.27 |
| *Proteobacteria* | *Unclassified* | *Unclassified* | -4.27 |
| *Actinobacteriota* | *Microbacteriaceae* | *Microbacterium* | -4.28 |
| *Actinobacteriota* | *Unclassified* | *Unclassified* | -4.28 |
| *Proteobacteria* | *Burkholderiaceae* | *Massilia* | -4.29 |
| *Proteobacteria* | *Burkholderiaceae* | *Unclassified* | -4.30 |
| *Bacteroidota* | *Flavobacteriaceae* | *Flavobacterium* | -4.33 |
| *Actinobacteriota* | *Microbacteriaceae* | *Mycetocola_A* | -4.34 |
| *Actinobacteriota* | *Microbacteriaceae* | *Leucobacter* | -4.34 |
| *Firmicutes_A* | *Clostridiaceae* | *Clostridium* | -4.35 |
| *Bacteroidota* | *Sphingobacteriaceae* | *Sphingobacterium* | -4.35 |
| *Proteobacteria* | *Pseudomonadaceae* | *Pseudomonas_E* | -4.35 |
| *Bacteroidota* | *Flavobacteriaceae* | *Flavobacterium* | -4.36 |
| *Proteobacteria* | *Beijerinckiaceae* | *Microvirga* | -4.41 |
| *Proteobacteria* | *Devosiaceae* | *Devosia* | -4.42 |
| *Proteobacteria* | *Shewanellaceae* | *Shewanella* | -4.43 |
| *Actinobacteriota* | *Mycobacteriaceae* | *Dietzia* | -4.45 |
| *Proteobacteria* | *Burkholderiaceae* | *Massilia* | -4.46 |
| *Proteobacteria* | *Moraxellaceae* | *Acinetobacter* | -4.47 |
| *Proteobacteria* | *Burkholderiaceae* | *Massilia* | -4.47 |
| *Planctomycetota* | *Isosphaeraceae* | *Singulisphaera* | -4.50 |
| *Actinobacteriota* | *Geodermatophilaceae* | *Modestobacter* | -4.51 |
| *Actinobacteriota* | *Micrococcaceae* | *Citricoccus* | -4.51 |
| *Proteobacteria* | *Sphingomonadaceae* | *Altererythrobacter_B* | -4.53 |
| *Actinobacteriota* | *Micrococcaceae* | *Arthrobacter_A* | -4.57 |
| *Bacteroidota* | *Weeksellaceae* | *Kaistella* | -4.58 |
| *Proteobacteria* | *Burkholderiaceae* | *Herminiimonas* | -4.60 |
| *Actinobacteriota* | *Microbacteriaceae* | *Unclassified* | -4.60 |
| *Proteobacteria* | *Acetobacteraceae* | *Paracraurococcus* | -4.61 |
| *Bacteroidota* | *Flavobacteriaceae* | *Flavobacterium* | -4.63 |
| *Proteobacteria* | *Acetobacteraceae* | *Paracraurococcus* | -4.64 |
| *Bacteroidota* | *Flavobacteriaceae* | *Flavobacterium* | -4.66 |
| *Proteobacteria* | *Burkholderiaceae* | *Albidiferax* | -4.67 |
| *Firmicutes* | *Carnobacteriaceae* | *Alkalibacterium* | -4.67 |
| *Actinobacteriota* | *Microbacteriaceae* | *Microbacterium* | -4.67 |
| *Actinobacteriota* | *Micrococcaceae* | *Arthrobacter_F* | -4.67 |
| *Deinococcota* | *Trueperaceae* | *Truepera* | -4.68 |
| *Proteobacteria* | *Moraxellaceae* | *Acinetobacter* | -4.69 |
| *Actinobacteriota* | *Nocardioidaceae* | *Nocardioides* | -4.69 |
| *Proteobacteria* | *Burkholderiaceae* | *Xylophilus* | -4.70 |
| *Proteobacteria* | *Burkholderiaceae* | *Janthinobacterium* | -4.72 |
| *Actinobacteriota* | *Propionibacteriaceae* | *NML-160184* | -4.74 |
| *Actinobacteriota* | *Microbacteriaceae* | *Root112D2* | -4.74 |
| *Bacteroidota* | *Spirosomaceae* | *Dyadobacter* | -4.79 |
| *Proteobacteria* | *Beijerinckiaceae* | *Bosea* | -4.81 |
| *Firmicutes* | *Planococcaceae* | *Rummeliibacillus* | -4.82 |
| *Bacteroidota* | *Flavobacteriaceae* | *Flavobacterium* | -4.82 |
| *Actinobacteriota* | *Microbacteriaceae* | *Unclassified* | -4.82 |
| *Proteobacteria* | *Burkholderiaceae* | *Massilia* | -4.82 |
| *Proteobacteria* | *Sphingomonadaceae* | *Sphingomonas* | -4.84 |
| *Proteobacteria* | *Enterobacteriaceae* | *Mixta* | -4.88 |
| *Proteobacteria* | *Acetobacteraceae* | *Gluconobacter* | -4.89 |
| *Firmicutes* | *Bacillaceae_D* | *Bacillus_H* | -4.90 |
| *Firmicutes* | *Planococcaceae* | *Planococcus* | -4.91 |
| *Cyanobacteria* | *Unclassified* | *Unclassified* | -4.95 |
| *Proteobacteria* | *Sphingomonadaceae* | *Sphingobium* | -4.98 |
| *Firmicutes* | *Lactobacillaceae* | *Pediococcus* | -5.00 |
| *Actinobacteriota* | *Geodermatophilaceae* | *Modestobacter* | -5.01 |
| *Actinobacteriota* | *Dermabacteraceae* | *Brachybacterium* | -5.03 |
| *Proteobacteria* | *Rhizobiaceae* | *Pararhizobium* | -5.03 |
| *Actinobacteriota* | *Microbacteriaceae* | *Naasia* | -5.04 |
| *Proteobacteria* | *Enterobacteriaceae* | *Mixta* | -5.04 |
| *Actinobacteriota* | *Unclassified* | *Unclassified* | -5.08 |
| *Actinobacteriota* | *Nakamurellaceae* | *Nakamurella* | -5.11 |
| *Bacteroidota* | *Flavobacteriaceae* | *Flavobacterium* | -5.12 |
| *Actinobacteriota* | *Microbacteriaceae* | *Microbacterium* | -5.12 |
| *Firmicutes* | *Turicibacteraceae* | *Turicibacter* | -5.14 |
| *Bacteroidota* | *Flavobacteriaceae* | *Flavobacterium* | -5.15 |
| *Bacteroidota* | *Flavobacteriaceae* | *Flavobacterium* | -5.17 |
| *Proteobacteria* | *Rhizobiaceae* | *Leaf454* | -5.19 |
| *Actinobacteriota* | *Cellulomonadaceae* | *Cellulomonas* | -5.21 |
| *Actinobacteriota* | *Microbacteriaceae* | *Unclassified* | -5.26 |
| *Proteobacteria* | *Burkholderiaceae* | *Pigmentiphaga* | -5.28 |
| *Proteobacteria* | *Caulobacteraceae* | *Caulobacter* | -5.29 |
| *Bacteroidota* | *Weeksellaceae* | *Chryseobacterium* | -5.30 |
| *Desulfobacterota* | *Unclassified* | *Unclassified* | -5.30 |
| *Bacteroidota* | *Sphingobacteriaceae* | *Pedobacter_B* | -5.31 |
| *Actinobacteriota* | *Microbacteriaceae* | *Unclassified* | -5.31 |
| *Actinobacteriota* | *Propionibacteriaceae* | *Tessaracoccus* | -5.32 |
| *Proteobacteria* | *Devosiaceae* | *Devosia* | -5.33 |
| *Proteobacteria* | *Caulobacteraceae* | *Caulobacter* | -5.33 |
| *Bacteroidota* | *Flavobacteriaceae* | *Flavobacterium* | -5.34 |
| *Firmicutes* | *Streptococcaceae* | *Streptococcus* | -5.34 |
| *Proteobacteria* | *Sphingomonadaceae* | *Elkelangia* | -5.35 |
| *Proteobacteria* | *Beijerinckiaceae* | *Bosea* | -5.35 |
| *Proteobacteria* | *Pseudomonadaceae* | *Pseudomonas_M* | -5.36 |
| *Firmicutes* | *Marinococcaceae* | *Marinococcus* | -5.38 |
| *Actinobacteriota* | *Propionibacteriaceae* | *Propionibacterium* | -5.39 |
| *Bacteroidota* | *Sphingobacteriaceae* | *Pedobacter* | -5.41 |
| *Proteobacteria* | *Burkholderiaceae* | *Janthinobacterium* | -5.46 |
| *Proteobacteria* | *Burkholderiaceae* | *Pigmentiphaga* | -5.46 |
| *Actinobacteriota* | *Microbacteriaceae* | *Microbacterium* | -5.47 |
| *Bacteroidota* | *Spirosomaceae* | *Dyadobacter* | -5.47 |
| *Firmicutes* | *Planococcaceae* | *Planococcus* | -5.52 |
| *Deinococcota* | *Deinococcaceae* | *Deinococcus* | -5.57 |
| *Actinobacteriota* | *Microbacteriaceae* | *Glaciihabitans* | -5.57 |
| *Actinobacteriota* | *Micrococcaceae* | *Unclassified* | -5.58 |
| *Proteobacteria* | *Burkholderiaceae* | *Polaromonas* | -5.58 |
| *Firmicutes* | *Carnobacteriaceae* | *Marinilactibacillus* | -5.59 |
| *Actinobacteriota* | *Microbacteriaceae* | *Microbacterium* | -5.59 |
| *Bacteroidota* | *Sphingobacteriaceae* | *Pedobacter* | -5.60 |
| *Proteobacteria* | *Burkholderiaceae* | *Polaromonas* | -5.60 |
| *Firmicutes_A* | *Clostridiaceae* | *Clostridium_AD* | -5.61 |
| *Proteobacteria* | *Enterobacteriaceae* | *Pectobacterium* | -5.62 |
| *Proteobacteria* | *Sphingomonadaceae* | *LB1R16* | -5.63 |
| *Proteobacteria* | *Beijerinckiaceae* | *Bosea* | -5.71 |
| *Proteobacteria* | *Pseudomonadaceae* | *Pseudomonas_E* | -5.72 |
| *Proteobacteria* | *Burkholderiaceae* | *Unclassified* | -5.75 |
| *Actinobacteriota* | *Mycobacteriaceae* | *Williamsia* | -5.76 |
| *Chloroflexota* | *Thermomicrobiaceae* | *Nitrolancea* | -5.77 |
| *Proteobacteria* | *Xanthobacteraceae* | *Tardiphaga* | -5.77 |
| *Bacteroidota* | *Sphingobacteriaceae* | *Pedobacter* | -5.85 |
| *Bacteroidota* | *Spirosomaceae* | *Dyadobacter* | -5.87 |
| *Bacteroidota* | *Spirosomaceae* | *Dyadobacter* | -5.91 |
| *Proteobacteria* | *Enterobacteriaceae* | *Pectobacterium* | -5.94 |
| *Firmicutes* | *Planococcaceae* | *Planococcus* | -5.95 |
| *Proteobacteria* | *Burkholderiaceae* | *Albidiferax* | -5.96 |
| *Proteobacteria* | *Sphingomonadaceae* | *Unclassified* | -5.98 |
| *Proteobacteria* | *Caulobacteraceae* | *Caulobacter* | -6.01 |
| *Actinobacteriota* | *Mycobacteriaceae* | *Dietzia* | -6.05 |
| *Proteobacteria* | *Rhodobacteraceae* | *Unclassified* | -6.07 |
| *Actinobacteriota* | *Micrococcaceae* | *Arthrobacter_A* | -6.08 |
| *Proteobacteria* | *Burkholderiaceae* | *Polaromonas* | -6.13 |
| *Firmicutes* | *Aerococcaceae* | *Jeotgalibaca* | -6.18 |
| *Actinobacteriota* | *Microbacteriaceae* | *Unclassified* | -6.22 |
| *Actinobacteriota* | *Dermatophilaceae* | *Humibacillus* | -6.26 |
| *Proteobacteria* | *Devosiaceae* | *Devosia* | -6.31 |
| *Firmicutes* | *Lactobacillaceae* | *Levilactobacillus* | -6.32 |
| *Firmicutes* | *Planococcaceae* | *Planococcus* | -6.38 |
| *Actinobacteriota* | *Dermatophilaceae* | *Serinicoccus* | -6.40 |
| *Actinobacteriota* | *Nakamurellaceae* | *Nakamurella* | -6.40 |
| *Actinobacteriota* | *Microbacteriaceae* | *Glaciihabitans* | -6.43 |
| *Actinobacteriota* | *Mycobacteriaceae* | *Corynebacterium* | -6.49 |
| *Firmicutes* | *Lactobacillaceae* | *Leuconostoc* | -6.54 |
| *Firmicutes* | *Anoxybacillaceae* | *Anoxybacillus* | -6.54 |
| *Actinobacteriota* | *Microbacteriaceae* | *Microbacterium* | -6.73 |
| *Proteobacteria* | *Xanthobacteraceae* | *Bradyrhizobium* | -6.98 |
| *Firmicutes* | *Enterococcaceae* | *Enterococcus* | -7.10 |
| *Bacteroidota* | *Weeksellaceae* | *Kaistella* | -7.18 |
| *Actinobacteriota* | *Beutenbergiaceae* | *Miniimonas* | -7.25 |
| *Actinobacteriota* | *Micrococcaceae* | *Citricoccus* | -7.36 |
| *Proteobacteria* | *Burkholderiaceae* | *Albidiferax* | -7.66 |
| *Actinobacteriota* | *Actinomycetaceae* | *ZLJ0423* | -8.02 |
| *Proteobacteria* | *Rhodobacteraceae* | *Cypionkella* | -8.35 |
| *Actinobacteriota* | *Microbacteriaceae* | *Naasia* | -8.65 |
| *Proteobacteria* | *Methylophilaceae* | *Methylotenera_A* | -8.66 |
| *Actinobacteriota* | *Propionibacteriaceae* | *Propionibacterium* | -9.70 |
| *Actinobacteriota* | *Propionibacteriaceae* | *Tessaracoccus* | -21.26 |
| *Proteobacteria* | *Rhodobacteraceae* | *Unclassified* | -21.38 |
| *Proteobacteria* | *Moraxellaceae* | *Psychrobacter* | -23.49 |
| *Proteobacteria* | *Burkholderiaceae* | *Rugamonas* | -23.98 |
| *Proteobacteria* | *Sphingomonadaceae* | *Polymorphobacter* | -24.18 |
| *Actinobacteriota* | *Nakamurellaceae* | *Nakamurella* | -24.64 |
| *Proteobacteria* | *Caulobacteraceae* | *Brevundimonas* | -25.09 |
| *Bacteroidota* | *Spirosomaceae* | *Dyadobacter* | -25.12 |
| *Proteobacteria* | *Xanthobacteraceae* | *Bradyrhizobium* | -25.71 |
| *Bacteroidota* | *Chitinophagaceae* | *Lacibacter* | -26.05 |
| *Proteobacteria* | *Pseudomonadaceae* | *Pseudomonas_E* | -26.46 |


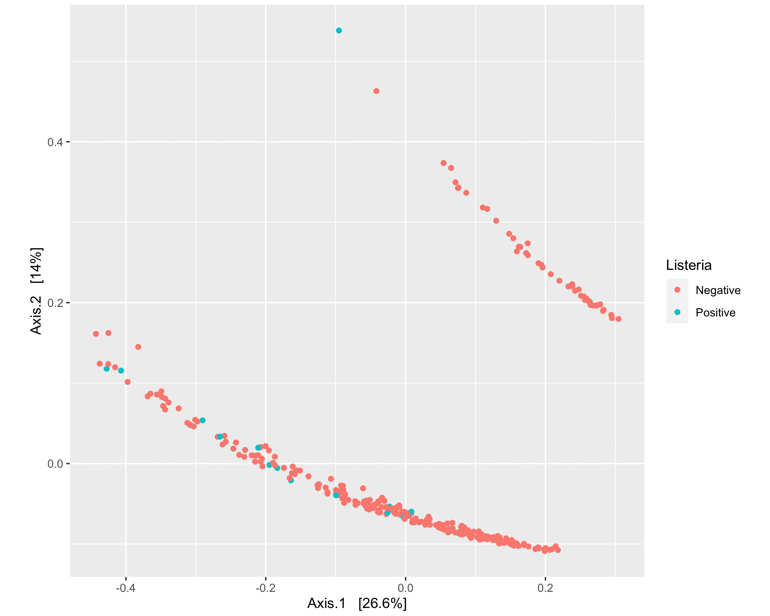

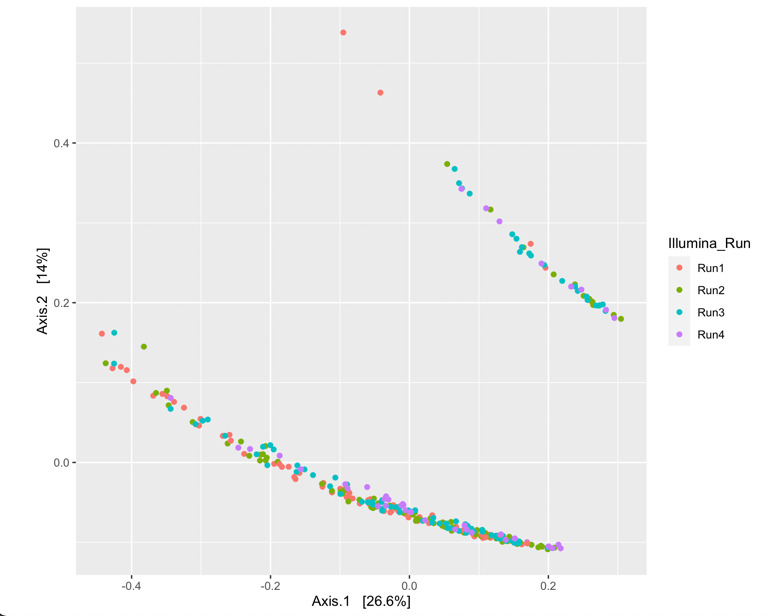

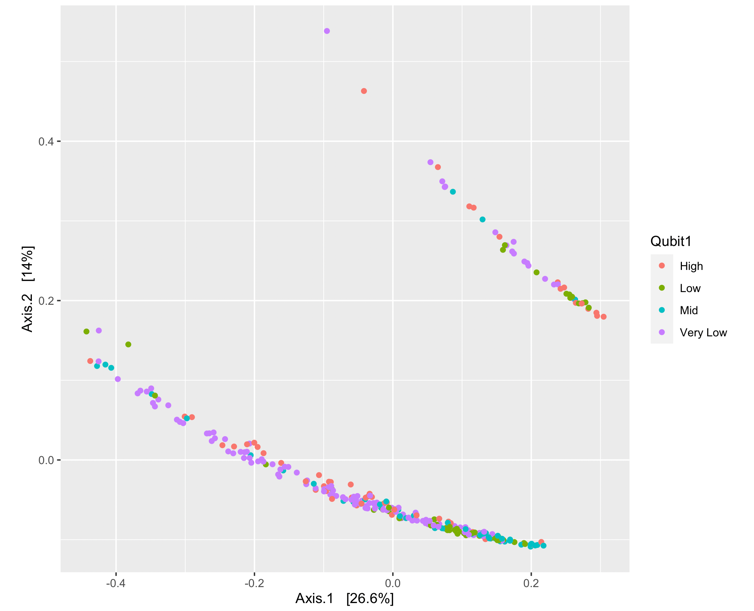

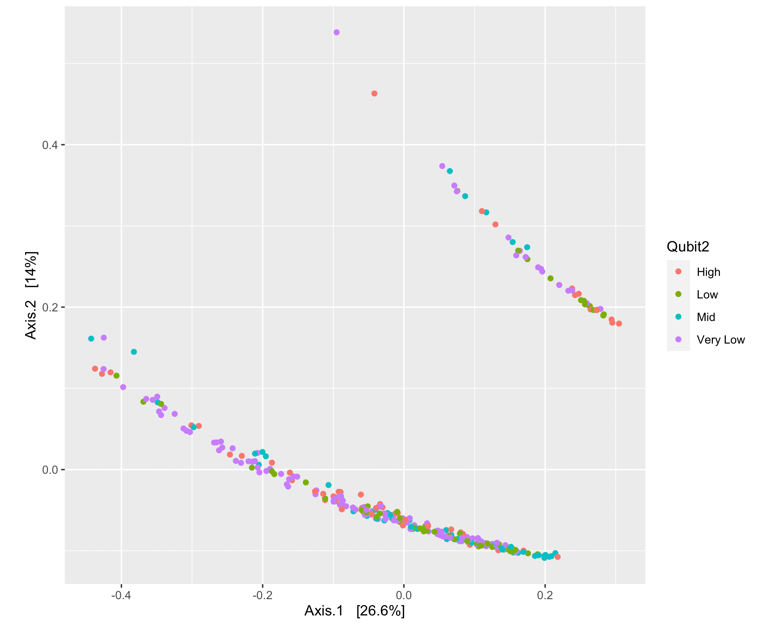


**A**

**B**

**C**

**D**

**Figure S1.** Selected PCoA plots against A) Illumina sequencing run, B) corresponding *Listeria* microbiological sample, C) Qubit concentrations pre-PCR, and D) Qubit concentrations post-PCR. Qubit concentration cut-offs were determining using quartile ranges.
